# A Class 1 Histone Deacetylase as Major Regulator of Secondary Metabolite Production in *Aspergillus nidulans*

**DOI:** 10.3389/fmicb.2018.02212

**Published:** 2018-09-19

**Authors:** Angelo Pidroni, Birgit Faber, Gerald Brosch, Ingo Bauer, Stefan Graessle

**Affiliations:** Division of Molecular Biology, Medical University of Innsbruck, Innsbruck, Austria

**Keywords:** histone deacetylases, secondary metabolites, histone modifications and chromatin structure, transcription factors, filamentous fungi, *Aspergillus*, antifungals

## Abstract

An outstanding feature of filamentous fungi is their ability to produce a wide variety of small bioactive molecules that contribute to their survival, fitness, and pathogenicity. The vast collection of these so-called secondary metabolites (SMs) includes molecules that play a role in virulence, protect fungi from environmental damage, act as toxins or antibiotics that harm host tissues, or hinder microbial competitors for food sources. Many of these compounds are used in medical treatment; however, biosynthetic genes for the production of these natural products are arranged in compact clusters that are commonly silent under growth conditions routinely used in laboratories. Consequently, a wide arsenal of yet unknown fungal metabolites is waiting to be discovered. Here, we describe the effects of deletion of *hosA*, one of four classical histone deacetylase (HDAC) genes in *Aspergillus nidulans*; we show that HosA acts as a major regulator of SMs in *Aspergillus* with converse regulatory effects depending on the metabolite gene cluster examined. Co-inhibition of all classical enzymes by the pan HDAC inhibitor trichostatin A and the analysis of HDAC double mutants indicate that HosA is able to override known regulatory effects of other HDACs such as the class 2 type enzyme HdaA. Chromatin immunoprecipitation analysis revealed a direct correlation between *hosA* deletion, the acetylation status of H4 and the regulation of SM cluster genes, whereas H3 hyper-acetylation could not be detected in all the upregulated SM clusters examined. Our data suggest that HosA has inductive effects on SM production in addition to its classical role as a repressor via deacetylation of histones. Moreover, a genome wide transcriptome analysis revealed that in addition to SMs, expression of several other important protein categories such as enzymes of the carbohydrate metabolism or proteins involved in disease, virulence, and defense are significantly affected by the deletion of HosA.

## Introduction

In addition to their indispensable ecological role in the recycling of organic material, filamentous fungi produce of a variety of commercially used compounds such as pigments, polysaccharides, vitamins, organic acids, enzymes, and even foodstuff such as miso, sake, shoyu, or Quorn (Newman and Cragg, 2012; Harvey et al., 2015). The most interesting fungal products, however, are small bioactive molecules that aid these organisms to adapt to adverse environmental conditions or to repel predators or microbes competing for food sources or habitats. Some of these molecules are dreaded mycotoxins that, if spoiled food is consumed, have a variety of detrimental effects on humans, ranging from allergic reactions and symptoms of poisoning to the triggering of cancer, if a low-dose exposure occurs over a longer time period (Pitt, 2000). Other SMs, however, are important as drugs against bacterial or fungal infections, hypertension, migraine, rejection of transplanted organs, heart disease, and as cholesterol-lowering substances (Fox and Howlett, 2008). Since only a minority of these molecules is produced under standard laboratory conditions, fungal species are representing a comprehensive source of a multitude of potential useful SMs that remain to be discovered. For instance, the genome of the mold *Aspergillus nidulans* holds putative genetic information for production of at least 30 still unknown secondary metabolites (Yaegashi et al., 2014) and thus, mining for novel beneficial substances in *Aspergillus* ssp. and many other fungal species is a major goal of mycologists worldwide. In the last decade, a huge number of strategies for the discovery of novel natural products of fungi have been developed. Among these strategies are optimized purification and screening techniques (e.g., Vansteelandt et al., 2013), a more sensitive detection of fungal metabolites (e.g., Klitgaard et al., 2015), and the application of microflow NMR coupled to untargeted mass spectrometry for structural identification of purified products (e.g., Bertrand et al., 2013). The fact that genes encoding enzymes involved in the biosynthesis of SMs such as polyketide synthases (PKSs), nonribosomal peptide synthetases (NRPSs), or terpene cyclases (TCs) are clustered on fungal chromosomes, does not only allow for their concerted regulation (Palmer and Keller, 2010) but also facilitates their detection from sequenced fungal genomes *in silico*, independently of the activity of certain SM clusters (e.g., van der Lee and Medema, 2016). Moreover, silent clusters can be induced by co-culturing of fungal strains together with another microorganism (e.g., Netzker et al., 2015) or via overexpression of specific transcription factors stimulating one or even more SM clusters. The latter approach led to the characterization of several novel metabolites in *Aspergillus* and several other species (e.g., Bergmann et al., 2007; Chiang et al., 2009; Chooi et al., 2013). An overview summarizing the multitude of emerging tools and technologies developed for the discovery of novel SMs was recently presented by Hautbergue et al. (2018) in a comprehensive review.

In recent years, however, also so-called “broad-domain regulators” such as LaeA (Bok et al., 2006), LaeB (Lin et al., 2018), or histone modifying enzymes (Shwab et al., 2007; Studt et al., 2016) were found to significantly impact the production of natural products in fungi. As in higher eukaryotes, genomic DNA of fungi is organized as chromatin and the extent of chromatin condensation determines the accessibility of genes or gene clusters for transcription factors. A complex combination of distinct histone modifications, termed as “histone code,” contributes to structural changes of chromatin structure and thereby regulates the readout of the genetic information (Strahl and Allis, 2000). Among these modifications, acetylation of the N-terminal core histone tails is of utmost importance. The dynamic process of acetylation involves two groups of enzymes: histone acetyl transferases (HATs) that transfer acetyl groups from acetyl-CoA to the ε-amino group of lysine residues and histone deacetylases (HDACs) that catalyze the removal of this modification and ensure the sensitive balance between acetylated and non-acetylated lysine residues of histones (Loidl, 1994; Verdin and Ott, 2015). Acetylation, however, not only acts as a specific signal on histones for the recruitment of distinct transcription factors (Loidl, 1994), but actually, those factors themselves may be substrates of HATs and HDACs (Martínez-Balbás et al., 2000). The fact that several HDACs have also been identified in bacteria and archaea further indicates that these enzymes represent an evolutionary ancient protein family that access non-histone targets as well (Leipe and Landsman, 1997).

*Aspergilli* and other filamentous fungi exhibit genes encoding between two and four NADP^+^-dependent “sirtuin” type HDACs, summarized as class 3 enzymes (Brosch et al., 2008) and four so-called “classical HDACs” homologous to the class 1 enzymes RpdA and HosA (Graessle et al., 2000) and the class 2 enzymes HdaA and HosB, respectively (Trojer et al., 2003). Catalytic activity of the classical enzymes can be inhibited by a group of hydroxamate-containing molecules such as trichostatin A (TSA) and its FDA-approved derivatives belinostat, panobinostat, and vorinostat (Zhang et al., 2018). The significance of classical HDACs in gene regulation of higher eukaryotes has turned these inhibitors into a promising novel category of anti-cancer drugs with an increasing therapeutic potential also for the treatment of other diseases (Yoon and Eom, 2016). Since the class 1 enzyme RpdA was recently found to be essential for growth and sporulation of (pathogenic) filamentous fungi, HDAC inhibitors are currently also tested as antifungal substances for the treatment of invasive fungal infections in immunosuppressed patients (Tribus et al., 2010). Moreover, fungal HDACs and their inhibitors gained additional significance since only 2 years after its first characterization (Tribus et al., 2005), the class 2 enzyme HdaA was shown to be involved in the repression of certain SM clusters in *A. nidulans* (Shwab et al., 2007). This finding was confirmed for HdaA homologs in several other species (e.g., Lee et al., 2009) and led to the exploitation of HDAC inhibitors for the activation of cryptic SM clusters in *Aspergillus*, *Alternaria*, *Penicillium*, and *Cladosporium* (e.g., Shwab et al., 2007; El-Hawary et al., 2018). However, as most of these “pan-inhibitors” are affecting all classical HDACs, it is difficult to attribute an effect to a specific enzyme. In addition, HDAC inhibitors might cause severe growth retardation in several fungal species, due to inhibition of the essential class 1 enzyme RpdA (Bauer et al., 2016) that probably causes indirect effects on SM production. In contrast, analyses of specific HDAC deletion mutants do allow insights into the functional role of single enzymes and, with suitable multiple deletion strains, even into possible interactions between different HDACs or HDAC classes.

We have previously demonstrated that deletion of the class 1 HDAC HDC1 of the maize pathogenic fungus *Cochliobolus carbonum* drastically reduced its virulence due to diminished expression of glucanase, polygalacturonase, and xylanase (Baidyaroy et al., 2001). Further analysis suggested that HDC1 might be directly involved in the activation of these genes, a previously unexpected and novel role of a histone deacetylase.

Here, we show that HosA, a so far not studied class 1 HDAC of *A. nidulans*, functions as both, an activator and a repressor of SM production. Our studies revealed that depletion of catalytic HosA activity supports the production of several novel bioactive molecules; in contrast others, most notably the antibiotic penicillin, are strictly dependent on HosA activity. Hence, HosA has, in addition to its classical role as a repressor, an activating function as well. Moreover, RNA-Seq analysis revealed that regulation of several other gene categories is affected by HosA. Among those categories are genes involved in carbon metabolism, disease, virulence and defense, and detoxification processes. However, in contrast to its yeast homolog HOS2 (Pfaller et al., 2009), HosA has no impact on fungal resistance against azole derivatives or other conventional antifungal substances.

## Materials and Methods

### Fungal Strains and Growth Media

Strains used in this study are listed in **Supplementary Table S1**. Strains were grown on glucose minimal medium (GMM) with appropriate supplerments as described (Cove, 1966). Xylanase promoter (*xylP*p) driven alleles were induced by addition of various concentrations (0.1–1%) of xylose to GMM (GMMX).

### Generation of Fungal Strains

Deletion of *hosA* was performed as described previously (Tribus et al., 2005). The selection marker *argB* was used for *hosA* deletion in strain A89. Positive *hosA* deletion strains were confirmed by PCR screening and single integration of the *hosA* deletion construct was verified by Southern analysis as described elsewhere (Graessle et al., 2000). For the complementation of *hosA*, the *hosA* delta strain TBF53.1 was co-transformed with a construct containing the *hosA* encoding sequence including the endogenous promoter and a plasmid comprising a phleomycin selection marker *bleR*, respectively (Austin et al., 1990). Two independent transformants (TBFl2.3 and TBFz7.1) that showed integration of the complementation construct at differing genetic loci were identified by Southern analysis and expression of *hosA* was verified via Northern analysis (data not shown).

Expression of His-, GFP-, and TAP-tagged (Bayram et al., 2012) HosA was achieved by an exchange of the endogenous *hosA* regulatory sequence for the inducible/repressible heterologous xylanase promoter (*xylP*p) of *Penicillium chrysogenum* as described previously (Tribus et al., 2010). Expression of His-tagged HosA in the strain A89 was performed by targeted integration of the expression construct at the *argB* locus as described in Lubertozzi and Keasling (2006). Three independent strains (TBF111, TBF117, and TBF122) carrying single integrations of the expression construct were identified by Southern and Northern blot analysis and expression of HosA was verified by immunoblot analysis using an anti-HosA antibody. For both, the GFP- and the TAP-tagged HosA expression, the *hosA* deletion strain TBF53.1 was used as recipient, and transformation was performed as co-transformation of the expression constructs together with a *bleR* carrying plasmid as described above. Expression of GFP- and TAP-tagged HosA was verified by immunoblot analysis using an anti-GFP and an anti-CBP antibody, respectively. Two independent expression strains of each of HosA-TAP (TBFXT1.1 and TBFXT3.1) and HosA-GFP (TBFGFP1.1 and TBFGFP4.1) were chosen for further analyses. For the expression of GFP-tagged RpdA, strain TIB54.1 was generated by targeted integration of pIB54 at the argB locus of strain A89 as described (Lubertozzi and Keasling, 2006).

### Sexual Crosses of *Aspergillus nidulans*

Generation of *hosA/hdaA* double knock out strains was performed as described previously (Todd et al., 2007) by crossing of a *hosA* deletion strain (TBF53.1) with a *hdaA* deletion strain (H4). Double mutant strains RBF115 and RBF117 were identified on selective media and verified by PCR.

### Antifungal Susceptibility Testing (ETEST^®^)

To compare drug susceptibility of *hosA* deletion and the wt strains ETEST^®^ (bioMérieux) was employed. ETEST^®^ consists of a predefined gradient of antifungal drugs on plastic strips. For this work ETEST^®^ strips containing fluconazole (SKU number 412349), voriconazole (SKU number 412489), amphotericin B (SKU number 526348), and caspofungin (SKU number 412268) were used. Strains were grown on GMM at 37°C for 48 h, spores were harvested and counted and a sterile swab was dipped into the spore suspension adjusted to 10^6^ conidia per ml. The inoculum was plated onto solid GMM and left to dry for 15 min at RT before the application of Etest strips^®^. Drug susceptibility of strains was evaluated after 24 h and 48 h of growth at 37°C.

### Northern and Western Analyses

Expression analyses of tagged HosA versions were performed under *xylP*p inductive (1% glucose/1% xylose) and repressive (1% glucose) conditions in minimal medium. RNA preparation, blotting, and hybridization were done as described (Graessle et al., 2000). Dig-dUTP-labeled DNA probes specific for the corresponding transcripts to be quantified were amplified with primers shown in **Supplementary Table S2**. Hybridized probes were detected by Anti-Digoxigenin-AP Fab fragments (Roche) and developed with CSPD chemiluminescent substrate (Roche) according to the manufacturer’s instructions. Signals were visualized by exposure to X-ray film or with the Fusion-SL 3500 WL imaging system (Vilber Lourmat). Total protein extracts were prepared by grinding 50–100 mg of lyophilized mycelia with a Tungsten Carbide ball in a mixer mill (Retsch^®^, MM 400) followed by extraction with 250–500 μl of buffer B250 as described in Bayram et al. (2012). Western blotting and detection was performed as described (Trojer et al., 2003). Proteins were detected by antibodies directed against HosA (Trojer et al., 2003) or anti-CBP (Millipore 07-482, 1:1333).

### Bioassay of Penicillin Activity

Bioassays were performed as described in Bok and Keller (2004) to determine penicillin (PN) activity. Fungal strains were grown in liquid medium, with an inoculum density of 5 × 10^6^ conidia per milliliter. After removal of the mycelium by filtration, the medium was lyophilized and resuspended in 1/5 volume of sterile *A. dest*. The PN sensitive *Kocuria rhizophila* strain ATCC9341 was grown in BD^TM^ BBL^TM^ Trypticase^TM^ Soy Broth until the culture reached an OD_600_ of 1.0. For the assay plates, 50 ml precooled (46°C) antibiotic medium 1 (Roth^®^) was mixed with 3.75 ml of *K. rhizophila* culture and poured into 14.5 cm petri dishes. Wells (Ø 8 mm) were pierced into the test plates and 100 μl of each sample were applied to wells. Control samples were pretreated with 15 U of penicillinase from *Bacillus cereus* (Sigma-Aldrich^®^). Assay plates were incubated at 4°C for 2 h, to allow initial diffusion of the samples and subsequently incubated over night at 37°C.

### Purification of HosA Activity and HDAC Assay

For purification of HosA-TAP, six 1000 ml Erlenmeyer flasks containing 200 ml each of GXMM media were inoculated with *A. nidulans* conidia (5 × 10^6^/ml) and incubated with shaking at 37°C for 15 h. Affinity purification until the first elution by TEV protease was performed as described (Bayram et al., 2012). Aliquots of the elution were directly used for HDAC assays or frozen in liquid nitrogen for storage at -80°C.

Enzymatic activity of enriched HosA was measured in triplicates using either [^3^H] acetate-prelabeled chicken histones as substrate (Trojer et al., 2003) or fluorometric labeled peptides of a commercial (EMD Millipore) HDAC Assay Kit according to the manufacturer’s instructions. Briefly, 15 μl of the IgG eluate were mixed with HDAC Assay Buffer containing either Trichostatin A [TSA] in several concentrations (5, 20, 100, 250, 500, and 750 nM), or DMSO as control and subsequently was incubated for 60 min at 25°C. After addition of 20 μl activator solution, samples were further incubated for 15 min at 20°C. Fluorescence was measured using a FLUOstar Omega Plate Reader (BMG Labtech) set to 355 and 460 nm for excitation and emission, respectively. Before use, the instrument was tested and calibrated by creating a standard curve as described in the protocol of the HDAC assay kit.

### Subcellular Localization of HosA

To determine the subcellular localization of HosA, the HosA-GFP expressing strain, was grown at 30°C overnight, on cover slips in six-well plates, under conditions of moderate (0.1% xylose) induction of the *xylP* promoter. As the cellular localization of RpdA was previously shown to be predominantly nuclear (Bauer et al., 2016), a strain expressing RpdA-GFP under the control of *xylP*p (TIB54.1) was used as reference. DNA was stained with DAPI.

### RNA-Seq Analysis

Strains were grown in triplicates in 100 ml of GMM at a density of 2 × 10^6^/ml at 37°C for 24 and 60 h, respectively. cDNA libraries of 12 samples were sequenced on the Illumina platform (Illumina HiSeq Single Read sequencing) in which reads of 50-bp were generated. The resulting reads were aligned using TopHat (version 2.1.1) (Trapnell et al., 2012) to the *A. nidulans* FGSC_A4 genome (version s10-m04-r06) received from the AspGD database. Gene expression levels were calculated with the Cufflinks package (version 2.2.1) and normalized by the number of fragments per kilobase of exon per million mapped reads (FPKM). Differential gene expression analysis was performed with Cuffdiff (version 2.2.1) (Trapnell et al., 2012). Tables containing expression values of each gene received from the Cuffdiff analysis were filtered (log2FC ≥ 2, FDR < 0.05) and prepared for MIPS functional catalog (FunCat) (Ruepp et al., 2004), ontology enrichment analysis by using the FungiFun2 platform (Priebe et al., 2015). FunCat categories with a false discovery rate (FDR) under 0.05 were defined as significantly enriched. The FDR correction of each directly and indirectly annotated top category was calculated using the Benjamini–Hochberg method. Data processing and generation of graphical plots were performed using R (version 3.5.0) with the packages dplyr (Wickham et al., 2017), ggplot2 (Wickham, 2009), pheatmap (Kolde, 2015), and VennDiagram (Chen, 2018). In general, schemes of SM gene clusters were generated using the illustrator for biological sequences (Liu et al., 2015).

### ChIP Analysis and qPCR

Chromatin immunoprecipitation analysis was performed as described (Boedi et al., 2012) with minor modifications. GMM medium was inoculated with *A. nidulans* conidia (10^6^/ml) and incubated at 30°C for 48 h. Crosslinking of proteins and DNA was induced by the addition of formaldehyde to a final concentration of 0.8% (w/v) and stopped with 750 mM TRIS-HCl [pH 8] after 15 min of further shaking. Chromatin of extracts of 300 mg of lyophilized mycelia was sheared in 1.5 ml-TPX tubes (Diagenode) using the Bioruptor plus (Diagenode) in cycles for 30 s “on” and 30 s “off” at maximal power. To produce soluble chromatin with average size between 200 and 600 bp, samples were sonicated between 5 and 10 cycles. After sonication tubes were centrifuged for 1 min at 15.000 × *g* at 4°C, supernatants were used in further steps. After pre-clearing, 300 μg of protein were incubated with 3 μg of antibody over night at 4°C on a rotary shaker. The protein-antibody conjugate was precipitated with 30 μl of paramagnetic Protein A beads (Dynabeads, Invitrogen) for 1 h at 4°C on a rotary shaker. Unspecific proteins were removed by two subsequent washes with 1 ml of washing buffer (0.2% SDS, 0.5% Triton X-100, 2 mM EDTA, 20 mM Tris-HCl pH 8) containing 150 mM NaCl and 500 mM NaCl, respectively. The chromatin-Dynabeads complex was washed twice in 1 ml of TE-buffer (1 mM EDTA, 10 mM Tris-HCl pH 8). For resuspension of the chromatin, 50 μl of fresh resuspension buffer (1% SDS, 0.1 M NaHCO_3_) was added, incubated at 65°C for 15 min, and centrifuged for 2 min. After repeating this step, supernatants were collected in a final volume of 100 μl. For reverse crosslinking, samples were incubated with 4 μl of NaCl (5 M) overnight at 65°C. After this step, 2 μl EDTA (0.5 M), 4 μl TRIS-HCl (1 M, pH 6.5), and 2 μl proteinase K was added and incubated for 1 h at 45°C. To remove RNA, samples were treated with RNase A (3% in total) and incubated at 65°C for 5 min and at 37°C for 30 min. DNA was purified with a PCR purification kit (QIAGEN) and eluted in 80 μl TE-buffer. For qPCR, DNA was used undiluted and in a 1:5 dilution whereas input controls were used in a 1:100 dilution.

Real-time PCR was performed with an ABI Prism 7900HT Detection System (Applied Biosystems). Standard curves were generated using eluted DNA from ChIP-experiments in serial dilutions. Two microliter of template was used in a 20-μl total volume reaction using Thermo Fisher Power SYBR^TM^ Green PCR master mix. PCR was performed in triplicates for each single ChIP experiment using primer pairs specific for three 5-prime regions of each of *orsA*, *ipnA*, *atnA*, and *cicB* (see **Supplementary Table S2**). To calculate the signal of enrichment for each region, percent of input normalization was used (100 × 2^∧^(Adj.input - Ct[IP])), whereas *Adj.input* = *Ct* (*input*) - log2 (*dilution factor*); average and standard deviation were generated from these values.

## Results

### *HosA* Deletion Causes Morphological Effects and Production of Pigments but Does Not Affect Resistance Against Antifungal Substances

In yeast and certain molds it was shown recently that the class 1 HDAC HOS2 contributes to resistance against antifungals such as voriconazole (Pfaller et al., 2009). This observation is of enormous medical interest, since efficacy of azoles used in antifungal therapies could be increased by combination with HDAC inhibitors, substances that recently gained importance as anti-cancer drugs (Dokmanovic et al., 2007). In order to investigate the role of HOS2-type enzymes in *Aspergilli*, the coding sequence of the HOS2 orthologous enzyme HosA was deleted. Spores of independent *hosA* deletion strains with a single genomic integration of the deletion construct were plated onto agar plates and ETEST strips (bioMérieux) loaded with increasing concentrations of the antifungal substances voriconazole, fluconazole, amphotericin B, and caspofungin, respectively, were applied to the plates. Deletion strains complemented for *hosA* were used as wildtype controls in the sensibility testing.

**FIGURE 1:**
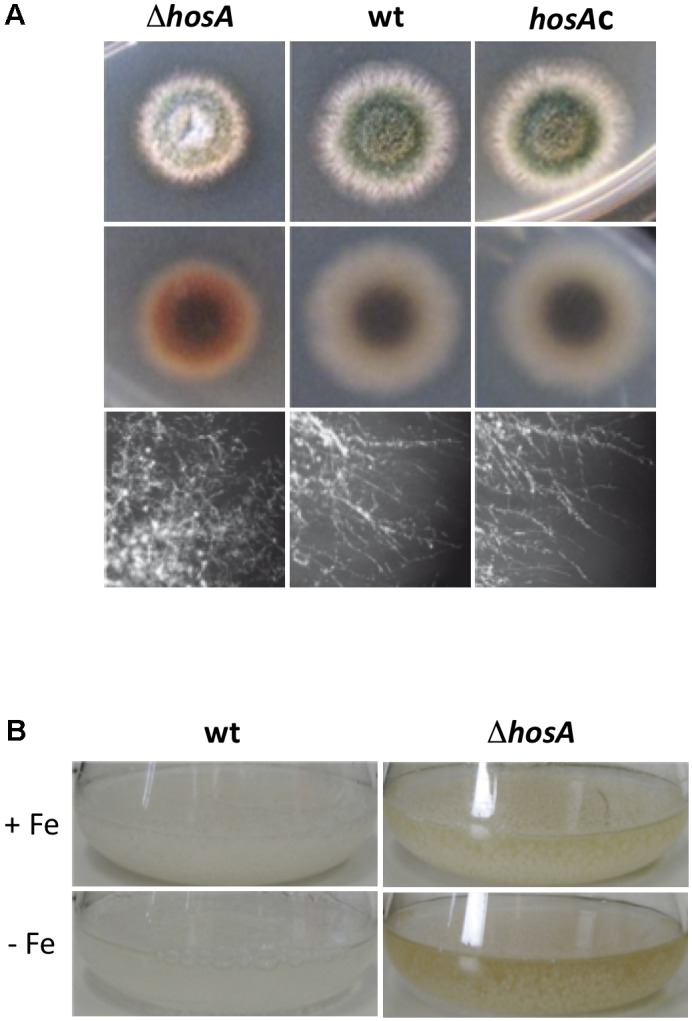
Phenotype of *hosA* knock out strains compared to wildtype and *hosA*-complemented controls. Strains were grown for 48 h at 37°C on minimal medium before colonies were assessed regarding to their size and color (top view and bottom view of the plates). Moreover, hyphal growth was microscopically surveyed at a magnification of 400× (lower row) **(A)**. Color of the growth medium of *hosA* mutants grown in submerged culture for 24 h in GMM with (-Fe) or without (+Fe) iron starvation was assessed and compared to wt cultures **(B)**.

Interestingly, examination of the ETEST plates after 48 h at 37°C revealed that *hosA* in *A. nidulans* did not lead to the expected increase of sensitivity against any of the antifungals tested. Indeed, sensitivity of the corresponding mutants against fluconazole seemed to be marginally decreased in some cases (**Supplementary Figure S1**). When spores were dotted onto plates without antifungal substances, however, growth and sporulation of colonies of the mutants were diminished and mycelia showed a significant reddish-brown coloration on the bottom side of the colonies (**Figure 1A**). Interestingly, a similar coloration was observed in the shaking culture supernatant of *hosA* delta strains grown for 24 h at 37°C (**Figure 1B**). Moreover, microscopic inspections revealed a significant hyper-branching of hyphae of the HosA mutants (**Figure 1A**). These results demonstrate that deletion of HosA does not alter the sensibility of *A. nidulans* to selected antifungal drugs but leads to a diminished growth and discoloration of hyphae and media.

### HosA Acts as Repressor of the Orsellinic Acid Gene Cluster

**FIGURE 2:**
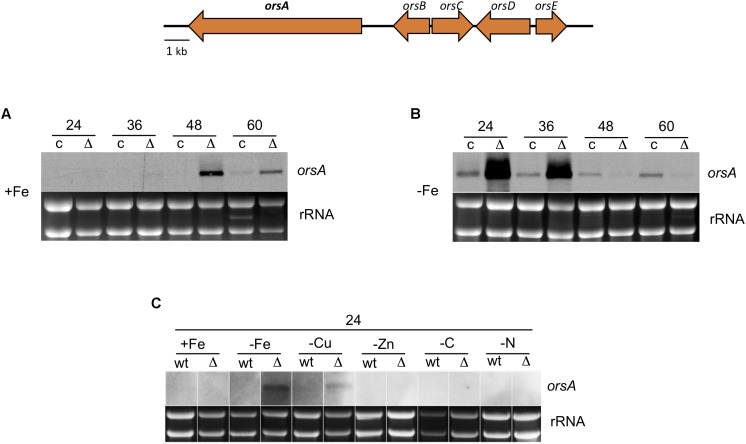
Expression of orsellinic acid biosynthetic cluster genes in *hosA* knock out strains and wildtype after different times of growth and under different starvation conditions. A schematic representation of the five cluster genes (*orsA-E*) is shown on the top of the figure, an *orsA* probe (bold) was used for Northern analysis. For *orsA* expression analysis, Δ*hosA* mutants and complemented strains were grown for 24, 36, 48 and 60 h at 37°C in a submerged culture (GMM) without **(A)** or with **(B)** iron starvation. Thirty micrograms of total RNA were botted, ethidium-stained rRNA was used as quality and loading control. *orsA* expression was also analyzed with 10 μg of RNA of *hosA* mutants and wt grown for 24 h under iron (-Fe), copper (-Cu), or zinc (-ZN) starvation and under carbon- (-C, 0.1% glucose) or nitrogen- (-N, 2 mM Gln) limitation **(C)**.

The striking pigmentation of HosA deletion strains on agar plates and in submerged culture suggested a possible role of HosA as repressor of secondary metabolites, as many of them are molecules contributing to the pigmentation of mycelia or are excreted to the culture medium. Two of such metabolites, the cathepsin K inhibitors F-9775A and F-9775B were identified recently as yellow-orange pigments of *A. nidulans.* Both compounds, however, are not produced under standard culture conditions but are induced in co-culture with the bacterial species *Streptomyces hygroscopicus* (Schroeckh et al., 2009) or under nitrate and orthophosphate limitation (Sarkar et al., 2012). Structural analyses of these two polyketides suggest that they are derivatives of the fungal archetypal polyketide orsellinic acid (OA). In order to prove a possible impact of HosA in the regulation of the OA biosynthetic gene cluster, *hosA* deletion strains were grown for 24, 36, 48, and 60 h and RNA was used for Northern blot analyses with a hybridization probe for *orsA*, a gene coding for a PKS of the OA biosynthetic gene cluster. After 36 h of growth, *hosA*-deleted strains showed weak and after 48 h strong transcription of *orsA*, whereas no transcription was detectable in the complemented control strains (**Figure 2A**). After 60 h of growth, however, a very weak transcript could be detected in the complemented strains as well, suggesting that not only deletion of *hosA* but also depletion of nutrients during growth might influence the regulation of the OA gene cluster. In order to prove this assumption, a HosA mutant and a wildtype strain were grown for 24 h under different starvation conditions. No effects were detected under zinc starvation or under carbon or nitrogen limitation (**Figure 2C**). Under copper or iron starvation, however, *orsA* transcription was moderately or considerably induced already in early *hosA* delta cultures and even in *hosA*-complemented strains and the wildtype, a weak *orsA* transcript was detected when 30 μg instead of 10 μg of RNA were loaded (**Figure 2B** and data not shown). These results demonstrate that transcription of *orsA* is induced under *hosA*-depleted conditions and is further enhanced under copper and iron starvation.

### Trichostatin A Effectively Inhibits Catalytic HosA Activity and Induces Expression of the Orsellinic Acid Biosynthetic Gene Cluster

**FIGURE 3:**
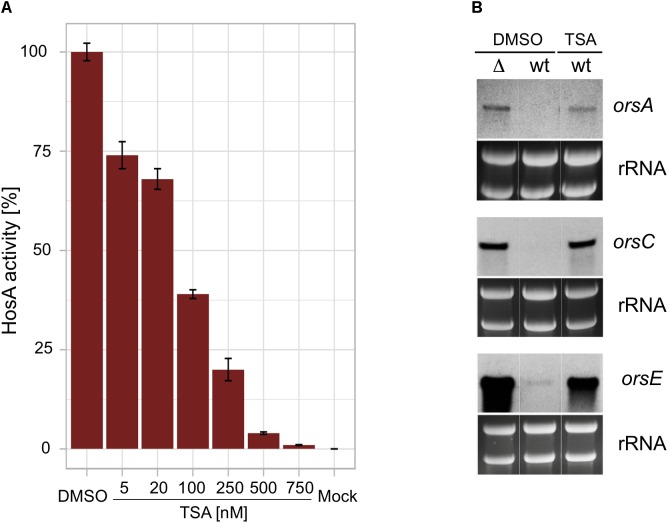
Effect of trichostatin A on the catalytic activity of HosA and the expression of orsellinic acid cluster genes. HDAC activity of purified TAP-tagged HosA was eluted by cleavage with TEV protease and aliquots were used for HDAC activity assays with increasing concentrations of the HDAC inhibitor trichostatin A (TSA). HosA activity with dimethyl sulfoxide (DMSO), the solvent of TSA, was set to 100%, an assay aliquot without recombinant HosA was used as mock control **(A)**. Expression of the orsellinic acid cluster genes *orsA*, *orsC*, and *orsE* in a wildtype strain with and without TSA treatment. *hosA* mutant and wt strains were grown in a submerged culture (GMM) for 21 h at 37°C under iron starvation before 1 μM TSA (or the corresponding amount of DMSO for the controls) was added to the cultures for additional 3 h. After harvesting of the strains, total RNA was prepared from the mycelia, blotted onto a Hybond-N membrane and hybridized with a digoxigenin-labeled probe against *orsA*, *orsC*, and *orsE*, respectively **(B)**.

Since OA expression was found to be stimulated in co-culture with *Streptomyces* strains (Schroeckh et al., 2009), bacteria known as producers of the HDAC inhibitor trichostatin A (TSA), we wondered, if OA biosynthetic cluster genes can be induced by TSA via the inhibition of HosA activity. In order to analyze the sensitivity of catalytic HosA activity to TSA, the enzyme was TAP-tagged as described (Bayram et al., 2012), expressed under the control of the heterologous xylanase promoter *xylP*p of *P. chrysogenum* in strain TBFXT1.1 (see **Supplementary Table S1**) and pulled down with IgG-Sepharose under native conditions. Eluted fractions were then analyzed by immunoblotting using an anti-CBP antibody as described (Bauer et al., 2016). Aliquots of enriched HosA were subsequently assayed for HDAC activity with increasing concentrations of TSA. Catalytic HosA activity was reduced by more than 60% when TSA was added to 100 nM final concentration. At 500 nM TSA, more than 95% of HosA activity was inhibited demonstrating its sensitivity to this inhibitor (**Figure 3A**).

This result prompted us to check the *in vivo* effect of TSA with regard to the transcription of the three OA cluster genes *orsA*, *orsC*, and *orsE*, respectively. To this end, an *A. nidulans* wild type was grown for 21 h in a submerged culture before 1 μM of TSA was added to the medium for 3 h. An untreated wildtype and a *hosA* delta strain were used as controls. RNA was prepared and analyzed with probes against the three OA cluster genes in a Northern blot. As shown in **Figure 3B**, treatment with TSA leads to an upregulation of the transcripts in the wildtype, very similar to that found in the *hosA* delta strain without TSA. No induction of the corresponding cluster genes was detectable in the wildtype control with DMSO. These results confirm a direct correlation between HosA activity and expression of OA biosynthetic cluster genes.

### Production of Penicillin Is HosA-Dependent

**FIGURE 4:**
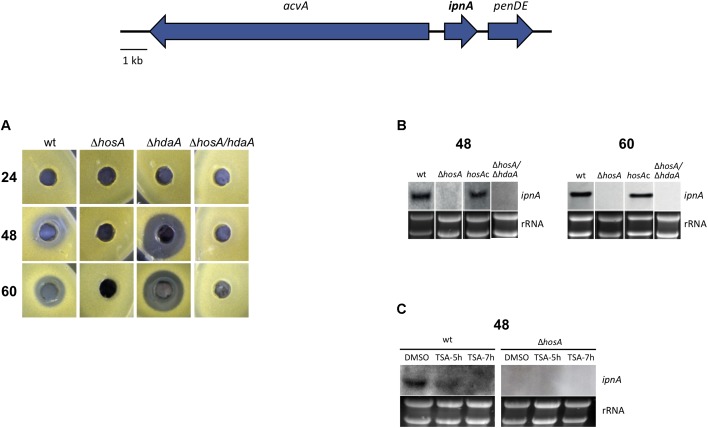
Production of penicillin and expression of the penicillin cluster gene *ipnA* in different HDAC mutants. A bacterial growth inhibition assay was used for quantification of penicillin production in *hosA-*, *hdaA-*, and *hosA/hdaA-*delta strains compared to wildtype. Strains were grown for 24, 48, and 60 h at 37°C in a submerged culture in GMM before media (sixfold concentrated) were pipetted into wells of agar plates inoculated with the PN sensitive bacteria strain *K. rhizophila* (ATCC9341). After incubation over night at 37°C, the size of the bacterial growth inhibition zone was analyzed. Bacterial clearing around the well corresponds to the relative production of penicillin of the strains tested **(A)**. Northern blots were used to verify the results of the bioassay. *hosA* and *hosA/hdaA* mutants, a complemented *hosA* mutant and wt were grown for 48 and 60 h at 37°C in submerged GMM cultures. RNA of strains was hybridized with a digoxigenin-labeled probe for the penicillin cluster gene *ipnA* (see schematic representation of the gene cluster on top of this figure). Ethidium-stained rRNA served as quality and loading control **(B)**. Impact of TSA on the penicillin production was assessed. *hosA* mutant and the wt strain grown for 43 and 41 h in GMM were treated with 1 μM TSA for 5 and 7 h. RNA was prepared, blotted, and hybridized with the *ipnA* probe. DMSO treated cultures served as negative controls **(C)**.

The fact that HosA activity represses OA biosynthesis prompted us to ask for the role of HosA for the production of other important SMs, such as penicillin (PN). In order to analyze if PN production is induced beyond wildtype level in *hosA* deletion mutants, a bacterial growth inhibition assay was performed as described (Shwab et al., 2007). Since we have shown that the class 2 type enzyme HdaA acts as repressor of PN production in *Aspergillus* (Shwab et al., 2007), we also were interested, if the induced PN production of the *hdaA* delta strain can be further increased by an additional deletion of *hosA*. To address both questions, we generated *hdaA/hosA* double knock out strains and analyzed them, together with the *hdaA* single knock out, in the bacterial growth inhibition assay. Compared to the wildtype control, the *hdaA* delta strain showed the expected increase in PN production after 48 and 60 h (**Figure 4A**). Unexpectedly, however, PN concentration in the growth medium of HosA mutants was not increased but decreased below the detection level of the bioassay. This rather surprising finding was also confirmed by results of the *hdaA*/*hosA* double mutants suggesting that the elimination of PN production by *hosA* deletion overrides PN upregulation caused by the deletion of *hdaA*. In order to verify these findings on the transcriptional level, Northern analysis was performed. The strain deleted for *hosA*, a *hosA*-complemented strain, an *hdaA/hosA* double mutant, and a wildtype control were grown for 48 and 60 h before RNA was prepared and hybridized with a probe against *ipnA*, encoding for isopenicillin N synthase. In contrast to the wildtype and the *hosA*-complemented strain that showed a clear *ipnA* transcript, no *ipnA* transcript was detectable in *hosA* and in *hdaA*/*hosA* mutants after 48 and 60 h (**Figure 4B**), confirming the findings of the bacterial growth inhibition assay. Hence, HosA is absolutely required for sufficient expression of *ipnA* in *A. nidulans*, even in the absence of HdaA.

The fact that a recent study revealed that class 1 HDAC complexes in yeast can promote nucleosome assembly independently of activity (Chen et al., 2012) raised the question, if HosA *per se* or its catalytic activity is required for the production of PN. To address this question, Northern analyses under TSA treatment were performed. A wildtype strain was grown for 43 or 41 h in submerged culture before 1 μM TSA was added to the medium for 5 or 7 h. A culture treated solely with the TSA solvent DMSO was used as negative control. After 5h of TSA treatment, *ipnA* transcription was significantly reduced compared to the control and after 7 h, transcription was beyond the detection level (**Figure 4C**). As expected, no transcript was detectable in a *hosA*-deleted control strain.

### HosA Overexpression Does Not Affect Penicillin Production but Leads to Minor Growth Retardation of the Expression Strains

Since HosA activity is obviously required for sufficient *ipnA* expression, we were interested whether HosA overexpression leads to PN levels beyond wildtype. In order to address this question, strains TBF117 and TBF122, expressing His-tagged HosA under *xylP*p (see **Supplementary Table S1**), were grown in submerged culture supplemented with 1% xylose or 1% glucose to induce or repress HosA expression. Crude protein extracts of strains were analyzed by immunoblotting using an anti-HosA antibody. In order to identify possible cross-reactions of the antibody with proteins of the crude extracts, a wildtype strain was used as negative control. Under repressive conditions, no signals were observed in the corresponding mutant strains, whereas a strong signal was detected under *xylP*p inductive conditions confirming an efficient and strong expression of recombinant HosA (**Supplementary Figure S2A**). As expected, no signal was detectable in crude protein extracts of the control strains due to the low expression levels of endogenous HosA (Trojer et al., 2003). RNA was prepared from the induced cultures and used for Northern analysis with a hybridization probe for *ipnA* as described above. Interestingly, transcription level of *ipnA* did not differ from that of a wildtype control suggesting that PN production is not increased over wildtype level under HosA overexpression (data not shown). However, on agar plates, overexpression of HosA led to a minor but significant retardation of colony growth when compared to wildtype and *hosA*-complemented strains (**Supplementary Figure S2B**).

### HosA Is a Major Regulator of Secondary Metabolites in *Aspergillus nidulans*

The significant but contrary results of *hosA* deletion on the production of OA and PN encouraged us to examine the effect of HosA on the transcriptome of *A. nidulans* with a specific focus on SM gene clusters. To this end, RNA from *hosA* delta mutants and complemented strains grown in liquid media for 24 and 60 h at 37°C was reversely transcribed, and cDNA was used for sequencing on an Illumina HiSeq platform. Three biological replicates were analyzed resulting in 12 RNA-Seq samples, a total of 714,529,862 high-quality short-sequence reads (approximately 50 bp) yielding 108 gb of transcriptomic sequence data. Almost 650 million short reads (90.9%) were successfully mapped to the *A. nidulans* reference genome (**Supplementary Table S3**). Raw sequencing data and processed files are accessible via gene expression omnibus (Barrett et al., 2013) provided by the NCBI (accession number GSE117388).

**FIGURE 5:**
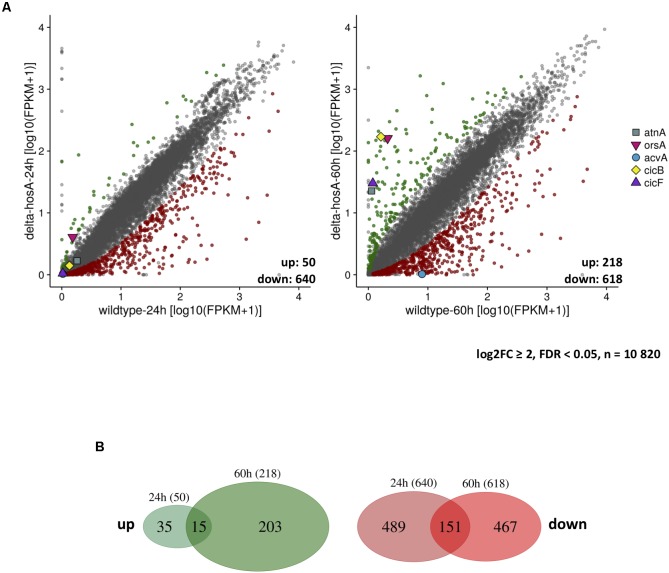
RNA-Seq analysis of differentially expressed genes in *hosA* mutants. Scatter plots show the expression values (log10[FPKM + 1]) for every annotated gene (*n* = 10.820) after 24 and 60 h of growth in GMM at 37°C. An additional cutoff (log2FC > 2/FDR < 0.05) for differentially expressed genes was applied. Dots within these cutoff criteria are displayed in gray and not considered as being affected by *hosA* deletion. Green dots indicate significantly upregulated, red dots significantly downregulated genes in *hosA* mutants. Biosynthetic cluster members of orsellinic acid (*orsA*), aspercryptin (*atnA*), cichorine (*cicB*, *cicF*), and penicillin (*acvA*) are highlighted, total number of up- and down-regulated genes are given **(A)**. Venn diagrams below illustrate the distribution of up- (green) and down-regulated (red) genes in the *hosA* mutants in 24 or 60 h cultures. Numbers of overlapping and non-overlapping up- and downregulated genes are show **(B)**.

Alignments were analyzed with Cufflinks (Trapnell et al., 2012) to conduct a genome-wide analysis of differential gene expression among *hosA* deletion strains and a wildtype control. Cuffdiff analysis revealed that 4,839 and 7,221 out of 10,820 annotated genes show a significantly different expression after 24 and 60 h of growth, respectively. To reduce this huge number of affected genes to the most significant ones, an additional cutoff (log2FC ≥ 2, FDR < 0.05) was applied resulting in 50 and 640 high-significantly up- and down-regulated genes in the *hosA* mutant after 24 h of growth. After 60 h, 218 and 618 genes were significantly up- and down-regulated (**Figure 5A** and **Supplementary Table S4**). Interestingly, only a minority of 15 up- and 151 down-regulated genes were overlapping in the short- and the long-term culture (**Figure 5B**).

**FIGURE 6:**
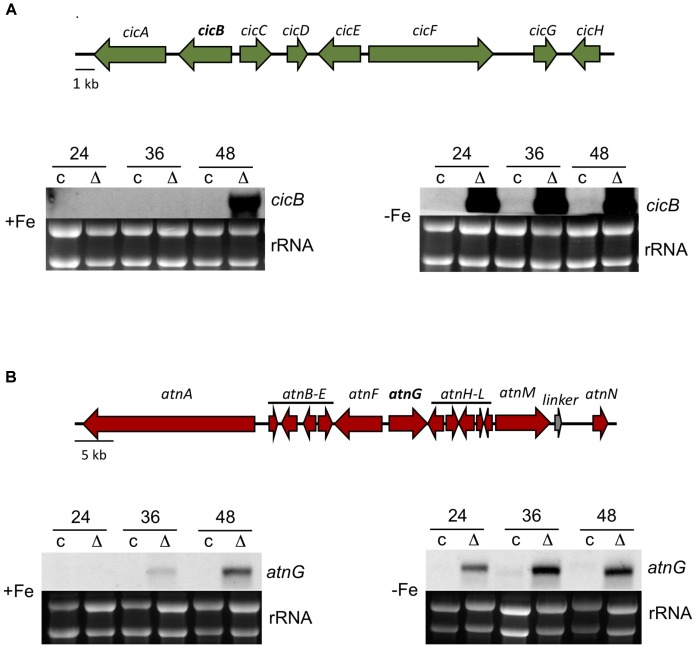
Expression analysis of members of the cichorine and aspercryptin biosynthetic gene cluster in *hosA* knock out mutants compared to *hosA*-complemented strains. A schematic representation of cluster genes of the cichorine- (*cicA-H*) and the aspercryptin- (*atnA-N*) gene cluster is shown on the top of the figure. For expression analysis, *hosA* mutants and complemented strains were grown for 24, 36, and 48 h at 37°C in GMM with (-Fe) or without (+Fe) iron starvation. Blotted RNA was hybridized with digoxigenin-labeled probes for the cichorine cluster gene *cicB* **(A)** and the aspercryptin cluster gene *atnG* **(B)**. Ethidium-stained rRNA was used as quality and loading control.

Among the down-regulated genes, also several SM cluster members showed diminished expression under *hosA* deletion. One of them, *acvA*, encodes the delta-(L-alpha-aminoadipyl)-L-cysteinyl-D-valine synthetase of the PN cluster, confirming the results of our bacterial growth assays and the Northern analysis (**Figure 4**). Other genes associated with categories related to secondary metabolism, however, were significantly upregulated (**Figure 5A**). In addition to the already identified *orsA* of the OA cluster (**Figure 2**), *cicB* and *cicF* of the cichorine (CC) cluster (Sanchez et al., 2012) and cluster members of novel secondary metabolites like aspercryptin (AC) (Chiang et al., 2016) were significantly upregulated by the deletion of *hosA*. To further confirm these results and to determine, if iron limitation leads to a similar enhancement of the HosA effect already shown for OA cluster genes, additional Northern experiments were performed with probes for *cicB* and *atnG*, two representatives of the CC and the AC cluster, respectively. Under iron sufficient conditions, *hosA* deletion strains showed a strong upregulation of both genes after 48 h, whereas no signals were detectable in the complemented controls (**Figure 6**). Under iron starvation, transcription of both, *cicB* and *atnG*, was already induced during an earlier stage of fungal growth, however, was exclusively observed in *hosA* delta strains, confirming the RNA-Seq results and the impact of iron limitation on the production of SMs.

**FIGURE 7:**
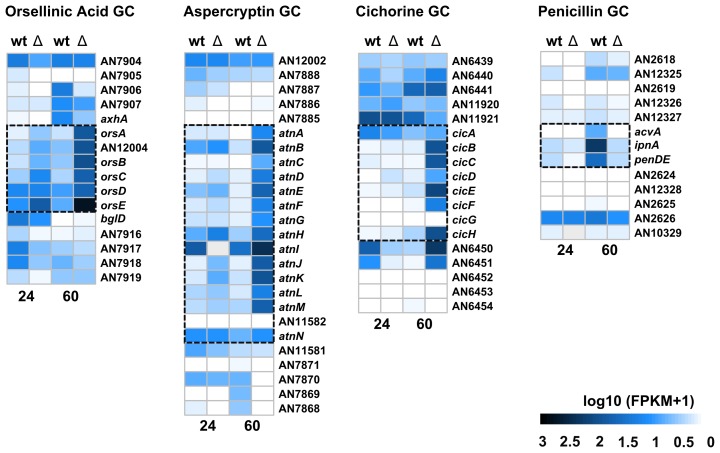
Expression analysis of the most affected biosynthetic clusters in *hosA* deletion mutants. Heatmaps display expression of the orsellinic acid, aspercryptin, cichorine, and penicillin secondary metabolite gene cluster members and adjacent genes after 24 and 60 h in *hosA* mutants versus wildtype strains. The predicted genes of each cluster are boxed, FPKM + 1 values are log10-transformed.

The significant role of HosA in the transcription of these SM cluster members prompted us to investigate, whether genes adjacent to the corresponding clusters are as well affected by the deletion of *hosA*. To address this question, RNA-Seq data of the four most prominently regulated SM clusters, OA, AC, CC, and PN, and five adjacent genes flanking both sides of each cluster were analyzed with regard to their transcription (**Supplementary Table S6**). Heatmaps of the cluster regions deduced from this analysis clearly illustrate that the regulatory effect of *hosA* deletion is almost exclusively restricted to these clusters (**Figure 7**). Adjacent genes were, if at all, only barely affected indicating a very specific HosA driven regulation.

In order to get an idea about the general impact of HosA on the regulation of SMs in *Aspergillus*, the expression values of 70 confirmed or putative coding sequences of proteins responsible for the biosynthesis of secondary metabolites (Khaldi et al., 2010; Nielsen et al., 2011; Andersen et al., 2013) were evaluated based on our transcriptome data (**Supplementary Table S5**). A heatmap deduced from this list clearly illustrates that a considerable number of these so called “key synthases” displays significant differences in expression level in wildtype and *hosA* deletion strains after 24 and/or 60 h (**Supplementary Figure S3**). Among these enzymes are members of well-known SM clusters such as StcJ, a putative PKS of the sterigmatocystin (ST) cluster (Brown et al., 1996; Bok et al., 2006), XptA, a prenyltransferase required for the prenyl xanthone synthesis (Sarkar et al., 2012), MicA, an NRPS involved in the production of microperfuranone (Yeh et al., 2012), and SidD, an NRPS involved in siderophore-mediated iron uptake (Schrettl et al., 2008). In order to determine the significance of HosA in the regulation of whole SM clusters, the expression of 20 adjacent genes up- and downstream of each key synthase was evaluated based on our transcriptome data. It turned out that expression of some clusters like the sterigmatocystin cluster was not collectively changed, as would have been suggested by StcJ. Other clusters, however, displayed significant regulation, although the corresponding key synthase was not affected by the deletion of *hosA*. Finally, expression values of 25 out of 70 predicted NRPSs and PKSs (listed in **Supplementary Table S5**) and their adjacent genes were selected for presentation. As shown in **Supplementary Table S7**, HosA affects in addition to the OA, AC, CC, and the PN cluster the transcription of several additional clusters such as the emericellamide (*easA/easB*) cluster (Chiang et al., 2008), the aspyridone (*apdA*) cluster (Bergmann et al., 2007), the asperfuranone (*afoE/afoG*) cluster (Chiang et al., 2009; Bergmann et al., 2010), and also cryptic clusters such as *pkhA/pkhB* and *inpA/inpB* (Ahuja et al., 2012; Inglis et al., 2013).

In summary, our RNA-Seq data revealed HosA as a major regulator of SMs of *A. nidulans* with converse regulatory effects depending on the metabolite gene cluster examined. This is substantiated by the finding that at least 14 already characterized SM gene clusters show significant differential regulation in the *hosA* mutants. Iron deficiency further increases the regulatory effects of *hosA* deletion and leads to a significantly earlier induction of the corresponding SM cluster genes.

### Chromatin Immunoprecipitation Analysis Suggests an Ambivalent Role of HosA in the Regulation of Fungal SMs

**FIGURE 8:**
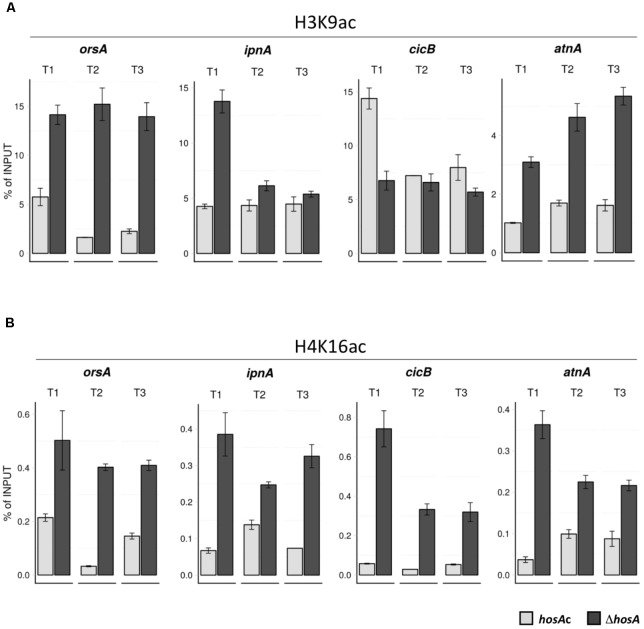
ChIP analysis of H3K9 and H4K16 acetylation in *hosA* mutants and *hosA*-complemented strains. Bars indicate enrichment of complemented strains (light) and *hosA* mutants (dark) in percentage of the input-DNA used for ChIP analysis. The crosslinked protein-DNA-complex was incubated with antibodies against H3K9ac **(A)** and H4K16ac **(B)**, respectively. For each of the genes *orsA*, *ipnA*, *cicB*, and *atnA*, qPCR was performed using primer-sets specific for three targets (T1, T2, T3) within the 5′ regions of the corresponding coding sequences. Error bars represent standard deviation calculated from triplets of each sample.

In order to confirm an effect of HosA on the acetylation status of SM cluster regions, chromatin immunoprecipitation (ChIP) analysis was established. Since acetylated (ac) H3K9 is closely correlated with active promoters (Karmodiya et al., 2012) and H4K16ac promotes chromatin compaction and transcriptional repression in yeasts (Kurdistani and Grunstein, 2003; Wirén et al., 2005; Abshiru et al., 2016), an anti-H3K9ac- and an anti-H4K16ac-antibody were chosen for the ChIP experiments. *hosA*-deleted mutants and *hosA*-complemented strains were grown for 48 h before formaldehyde was added to the shaking culture for crosslinking. After sonication, immunoprecipitation, reversed crosslinking, and protein and RNA digestion, purified DNA was used for quantitative PCR. To this end, three different 5-prime target regions (T1, T2, and T3, see **Supplementary Table S2**) of each of the cluster genes *orsA* (OA cluster), *ipnA* (PN cluster), *cicB* (CC cluster), and *atnA* (AC cluster), were considered for quantification. Interestingly, all cluster genes (including the repressed *ipnA*) showed significant hyper-acetylation of H4K16 under *hosA* deletion in all the regions analyzed (**Figure 8B**), while H3K9 hyper-acetylation was only detectable in *orsA* and *atnA* in all three regions. Only one region (T1) of *ipnA* showed increased acetylation of H3K9 (**Figure 8A**), whereas no significant acetylation beyond wildtype level was observed for *cicB*. Indeed, one region of *cicB*, T1, actually showed significant hypo-acetylation on H3K9 in *hosA* mutants.

Taken together, our results indicate an important role of HosA in H4K16 deacetylation linked to repression of prominent SM clusters such as OA, CC, and AC. The fact that *ipnA*, although repressed in *hosA* deletion strains, is as well hyper-acetylated at H4K16 suggests that HosA acts in addition as a dominating inductor of some gene clusters – an uncommon function that remains to be studied in more detail. In contrast, as shown for *cicB*, H3 hyper-acetylation is no absolute requirement for transcriptional activation of all gene clusters.

## Discussion

Filamentous fungi comprise several human pathogens that can cause serious infections such as invasive aspergillosis due to *Aspergillus* ssp., which may be severe and often life threatening in immunosuppressed patients (Lamoth et al., 2015). As key players in eukaryotic gene expression, HDACs are involved in the regulation of many proteins of fungi. Some of those represent virulence factors or essential molecules for fungal survival (Brosch et al., 2008). Inhibitors of HDACs, which are already exploited as potential therapeutic and chemo-preventive agents against cancer, neurodegenerative disorders, and graft intolerance, are also discussed as potential antifungal agents for the treatment of invasive fungal infections (Elaut et al., 2007). Due to limited efficiency of classical antifungal drugs and an increasing resistance against established azole derivatives, there is an urgent need for alternative therapy regimes. A few years ago, a novel antifungal substance, MGCD290 (MethylGene Inc., Montreal, QC, Canada), was identified as a specific HOS2 inhibitor in *Candida ssp.* (Pfaller et al., 2009). As an antifungal agent, MGCD290 alone displayed only moderate activity, however, in combination with azoles, it significantly increased the *in vitro* susceptibility of fungal species such as *Aspergillus fumigatus*, *Aspergillus flavus*, *Aspergillus niger*, and *Aspergillus terreus* (Lamoth et al., 2015).

These results prompted us to delete *hosA*, the gene for the HOS2 homologue of *A. nidulans*, a so far not studied class 1 HDAC in *Aspergilli*. Subsequently, HosA deficient mutant strains were analyzed with regard to their sensitivity against azoles and other established antifungal substances. In contrast to the increased sensitivity observed under MGCD290 treatment (Pfaller et al., 2009), *hosA* deletion did not affect the efficacy of any antifungal tested. These contradictory results may be explained by either a different biological function of HosA type proteins in different *Aspergillus* strains and species or, more likely, by the fact that MGCD290 is not exclusively acting on HosA type enzymes in filamentous fungi but influences the function of other classical HDACs as well. One of these HDACs, RpdA, was recently found to be required for viability of the opportunistic pathogen *A. fumigatus* (Bauer et al., 2016) and therefore, diminished RpdA activity due to MGCD290 treatment might potentiate the antifungal effect of azoles. Similar synergies between azoles and inhibition of HDACs were observed in some *Candida* species (Smith and Edlind, 2002). In these strains, TSA treatment led to a reduced up-regulation of multi drug transporter- (CDR) and azole target-(ERG) genes; it was speculated that TSA treatment is associated with histone acetylation of the promoter region of repressors of the CDR- and ERG-genes. The responsible HDAC, however, remained undiscovered. Our results suggest that in *A. nidulans*, HosA has neither direct nor indirect effect on drug susceptibility to azoles and thus cannot be regarded as a preferential target for inhibitors in order to enhance efficacy of conventional antifungal therapies.

However, HosA deficiency or its catalytic inhibition by TSA did show a remarkable effect on the transcription of various SM clusters and, most likely, on the production of the corresponding secondary metabolites of *A. nidulans*. In general, regulation of SM clusters in filamentous fungi is complex and involves multiple protein complexes (Brakhage, 2013). Many transcription factors (TFs) are thereby located on the corresponding gene cluster itself and are specifically regulated by various environmental stimuli. In addition, also trans-acting TFs exist that crosstalk between different clusters even on different chromosomes (Bergmann et al., 2010). In recent years, it turned out that important regulators such as LaeA as well as histone modifying enzymes are also able to regulate the production of several SMs (Bok and Keller, 2004; Gacek and Strauss, 2012). The class 2 HDAC HdaA of *A. nidulans* was the first example of such a histone modifying enzyme. In *hdaA* deletion strains, we could show a significantly increased production mainly of two secondary metabolites of *A. nidulans*: the carcinogenic aflatoxin precursor sterigmatocystin and the antibiotic penicillin (Shwab et al., 2007). Since addition of TSA to growth media of other fungal species such as *Alternaria* and *Penicillium* increased the production of several cryptic SMs as well, it was speculated that this upregulation is mainly due to the inhibition of the HdaA orthologues in these fungi (Shwab et al., 2007). The fact that HosA now has been proven as another major regulator of SMs in *Aspergillus* suggests, that the observed effects of HDAC inhibitors are most likely not exclusively due to HdaA inhibition. Indeed, our analysis of *hosA/hdaA* double mutants actually implies that the inductive effect on penicillin production via HdaA inhibition can even be overruled by the repressive effect of depleted HosA activity.

The finding that depletion (or inhibition) of an HDAC is silencing gene transcription is uncommon but not entirely unexpected. The first HDAC gene we have deleted in a filamentous fungus was *HDC1* in the plant pathogen *C. carbonum* (Baidyaroy et al., 2001). *HDC1* was also the first example for a significant downregulation of specific genes (encoding extracellular depolymerases) in an HDAC mutant, resulting in non-pathogenic *Cochliobolus* mutants. Subsequently, upregulation of specific genes via HOS2 type proteins was substantiated in *Saccharomyces cerevisiae* (Wang et al., 2002) and recent investigations in the phytopathogenic fungus *Fusarium fujikuroi* also confirmed a significant negative effect on both, pathogenicity and production of four SMs in strains with deleted *ffhda2*, a *hosA* homolog of this fungus (Studt et al., 2013). In these mutants, production of three secondary metabolites, gibberellin, bikaverin, and fusarin, was decreased between 65 and 80% and expression of fusaric acid was almost completely abolished resembling the situation of penicillin in *Aspergillus hosA* delta strains. A similar unexpected effect was also observed recently by Fan et al. (2017), where deletion of an HAT in the entomopathogenic fungus *Metarhizium robertsii* led to the characterization of not less than 11 new fungal metabolites confirming an inductive effect of hypo-acetylation on the production of certain SM clusters.

**FIGURE 9:**
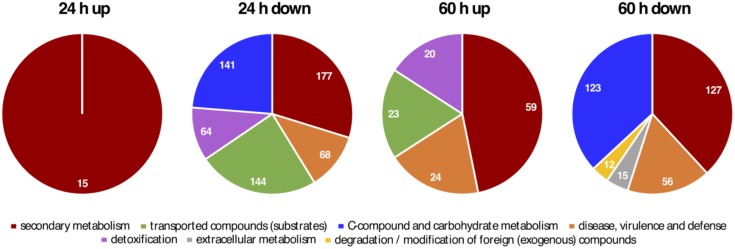
Most significantly enriched FunCat gene categories of *hosA* mutant strains versus wildtype. Differentially expressed genes in the RNA-Seq analysis were used for a MIPS Functional Catalogue (FunCat) ontology analysis on the FungiFun2 platform (also see **Supplementary Table S8**). Pie charts illustrate the most significantly enriched FunCat categories of down- and up-regulated genes after 24 and 60 h of growth from hierarchy level 2. Numbers of genes related to the significant categories are displayed within the pie charts.

In addition to its cryptic role as inducer of the penicillin cluster and several other genes associated to SM production or other protein categories (**Figure 9** and **Supplementary Table S8**), HosA as well functions as a repressor of SMs in *Aspergillus.* Although the regulatory principle of HosA remains to be studied in detail for each gene cluster affected, several possibilities are conceivable of how such opposing effects on transcription could be achieved by one and the same enzyme. HosA might directly deacetylate specific lysines on histones H3 and H4, repressing transcription of the respective gene-region, as shown for instance for orsellinic acid and aspercryptin cluster genes (**Figure 6**). Indeed, it has been demonstrated for the orsellinic acid cluster that H3K9 acetylation, mediated by the Saga/Ada complex, might trigger its activation (Nützmann et al., 2011) and a recent publication reports on two HATs of the plant pathogenic fungus *Fusarium graminearum* required for the regulation of secondary metabolism via acetylation of several lysine residues of H3 (Kong et al., 2018).

On the other hand, there is evidence from yeast that HOS2, in addition to its classical role as a repressor, might directly induce transcription by the deacetylation of (hyper-)acetylated H3 and H4 sites (Wang et al., 2002), a conceivable possibility why penicillin cluster genes such as *ipnA* are downregulated in *hosA* mutants. Alternatively, HosA might modify penicillin-specific TFs that induce genes independently of the acetylation level of the corresponding histones. It is known that probably all HDACs are able to target non-histone proteins such as transcriptional regulators as well (Glozak et al., 2005). The fact that catalytic activity of purified HosA is rather low when measured *in vitro* with acetylated chicken histones as substrate [(Trojer et al., 2003) and data not shown], but is significantly increased when acetylated peptides were used (**Figure 3**), strengthens the assumption that histones are not the predominate substrates of HosA. In addition, GFP-tagged HosA was detectable in both, the nucleus and the cytoplasm of the hyphae, also indicating additional non-histone targets of HosA (**Supplementary Figure S4**). This observation is in accordance with results from yeast, where HOS2 was shown to shuttle between the cytoplasm and the nucleus via a chaperone dependent process (Liu et al., 2012). Moreover, during the preparation of this manuscript, a paper was published that presented clear evidence that a HosA ortholog in the insect-pathogenic fungus *Beauveria bassiana* is indirectly involved in both, global acetylation and phosphorylation of H3K56 and H2AS129, respectively, providing further evidence of an indirect effect of HosA-type proteins in the regulation of fungal transcription via deacetylation of HATs or kinases (Cai et al., 2018).

Irrespective of evidence that HosA indirectly regulates SM production via activation of TFs or histone modifiers and directly via deacetylation of H3K9 and H4K16, we cannot exclude additional target sites on histones not addressed in our ChIP strategy. One out of further possible targets that was described to be hyper-acetylated under overexpression of the histone acetyltransferase EsaA is H4K12, leading to an increased transcription of SM clusters including the otherwise silent OA cluster (Soukup et al., 2012).

Interestingly, expression of silent SMs such as orsellinic acid, lecanoric acid, and the two yellow-orange cathepsin K inhibitors F-9775A and F9775B was also induced, when *A. nidulans* was grown in co-culture with the bacterial species *S. hygroscopicus* (Schroeckh et al., 2009). *Streptomyces* ssp. are known as natural producers of TSA suggesting that, in co-culture with *Aspergillus*, inhibition of HosA via bacterial TSA might add to the induction of the orsellinic acid cluster and, as shown here, also contributes to inhibition of penicillin production. Since antibiotics such as penicillin are natural fungal weapons against competing microorganisms, it is conceivable that their inhibition via secretion of HDAC inhibitors might be an appropriate biological answer of *Streptomyces* in a chemical warfare of microbes for limited resources.

Moreover, expression of orsellinic acid was also found under limitation of nitrogen and phosphorus (Sarkar et al., 2012). Our analysis revealed that alternative starvation conditions such as zinc, carbon, or nitrogen depletion do not affect orsellinic acid cluster genes with two exceptions, copper- and, more striking, iron-starvation. Both conditions significantly triggered orsellinic acid expression and, to a lower extent, that of other SMs in wildtype strains as well (**Figure 2C**). In addition, iron limitation showed also synergistic effects with *hosA* depletion, further increasing the regulatory effect of the *hosA* mutants. Although the specific interplay between HosA and iron remains to be discovered in detail, RNA-Seq data analysis (focused on genes responsible for fungal iron acquisition) revealed that *sidD*, encoding an NRPS required for the biosynthesis of triacetyfusarinine C (TAFC), is among the genes that are suppressed in the *hosA* mutants (**Supplementary Figure S3**). Since TAFC is essential for the synthesis of extracellular siderophores required for the mobilization of extracellular iron under iron starvation (Schrettl et al., 2008), it is plausible that diminished availability of intracellular iron contributes to the observed transcriptional effects of the SM clusters in *hosA* delta strains. Indeed, an iron-depending production of SMs was quite recently demonstrated for *ppzA* deletion strains of *A. fumigatus. ppzA* codes for the catalytic subunit of a protein phosphatase (PpZ) that was discussed as another epigenetic regulator of chromatin structure of the gene clusters affected (Manfiolli et al., 2017). The impact of PpZ on the production of SMs such as fumiquilazoline A, fumagillin, and helvolic acid was as well depending on the disposability of iron. PpzA thereby perturbed the response to iron assimilation of the fungus affecting its siderophore production. Moreover, data of other groups imply that several SMs are subject to iron-dependent regulation by SreA and HapX, two major transcription factors of iron homeostasis, confirming its importance for the production of fungal metabolites (Wiemann et al., 2014).

In addition to the SMs discussed in detail above, our experiments revealed that several other clusters are specifically affected in *hosA* mutants. Among them the cichorine cluster, responsible for the production of a phytotoxin (Sanchez et al., 2012) and the aspercryptin cluster, responsible for the production of a lipoprotein family. The latter was discovered quite recently by an HDAC inhibitor-based strategy (Chiang et al., 2016). Interestingly, two of these lipoproteins, aspercryptin A1 and B2, were identified and characterized by comparative MS analysis and extensive NMR of a wildtype strain versus an RpdA knock-down mutant (Henke et al., 2016). Moderate depletion of this essential class 1 HDAC thereby led to hyper-acetylation of bulk chromatin increasing the expression of these two metabolites. This observation suggests that aspercryptins are silenced by RpdA as well. Unfortunately, however, nothing is known about the impact of RpdA on other HosA-affected (cryptic) SM clusters. One of those clusters is the INP cluster, comprising seven genes (AN3490 to AN3496) on chromosome II. Although silent under laboratory growth conditions, the corresponding metabolite could be characterized by overexpressing its internal regulator *scpR* (Bergmann et al., 2010). Interestingly, *scpR* overexpression led to a regulatory crosstalk inducing another cluster on chromosome VIII responsible for asperfuranone (AF) biosynthesis. Although our *hosA* deletion strains showed an early and strong induction of all seven INP cluster genes, the AF cluster remained unaffected at 24 h of growth. After 60 h, however, the AF cluster genes *afoE* and *afoG* and their activator *afoA* were induced in *hosA* mutants suggesting, that induction of the AF cluster by *scpR* occurs with a time delay.

In 24 h cultures, another cryptic SM cluster (spanning genes AN2030 to AN2036) was significantly upregulated in *hosA* mutant strains. Although two of these genes, *pkhA* (AN2032) and *pkhB* (AN2035), could be characterized by overexpression as PKS encoding sequences, the final products of this cluster are waiting to be discovered (Ahuja et al., 2012), illustrating an example, how deletion of *hosA* might contribute to the characterization of still unknown compounds of filamentous fungi.

Very recently, a few other TFs were identified that, when deleted, resulted in induction or repression of SM production. For instance, inactivation of three novel regulators, LaeB, SntB, and HamI, resulted in a complete loss of aflatoxin production in *A. flavus* (Pfannenstiel et al., 2017), whereas deletion of *mcrA*, a gene encoding for a multicluster regulator present in several fungal species, induced the production of at least 10 small bioactive molecules and allowed the identification of three unknown compounds of *A. nidulans* (Oakley et al., 2016). Nevertheless, data revealed that McrA and a second major regulator of SMs in fungi, LaeA (Bok and Keller, 2004), are affecting fewer than 50% of the SM gene clusters known, indicating that several other important key players are involved in the complex regulation of fungal SMs. Our data suggest that HosA actually is one of those players.

## Conclusion

In conclusion, our results have revealed HosA as a novel master regulator of SMs in *A. nidulans* that, when deleted or inhibited, represents a promising possibility to open the fungal portfolio rife with bioactive molecules for possible medical applications.

## Author Contributions

AP, BF, and IB generated the data. SG, GB, and IB conceived and designed the experiments. IB, AP, and SG analyzed the data. SG, BF, AP, and IB wrote the manuscript.

## Conflict of Interest Statement

The authors declare that the research was conducted in the absence of any commercial or financial relationships that could be construed as a potential conflict of interest.

## Supplementary Material

FIGURE S1 Sensibility of *hosA* mutants compared to complemented strains as tested by antifungigrams (ETEST). For sensibility testing against antifungal substances, ETEST strips loaded with an increasing concentration of the antifungals voriconazole, amphotericin, caspofungin, and fluconazole were applied to plates with spores of *hosA* deletion strains and wt according to the manufacturer’s instructions of the test-strips. After incubation for 24 (not shown) and 48 h at 37°C, the sensibility was evaluated (see magnification of the fluconazole strip below).

FIGURE S2 Phenotypical analysis of HosA overexpression strains. His tagged HosA was overexpressed in *Aspergillus nidulans* strains TBF117 (*hosA*oe1) and TBF122 (*hosA*oe2) under control of the heterologous xylanase promoter (*xylP*p) in submerged culture (GMM) supplemented with 1% xylose (i) for 48 h at 37°C. In order to verify HosA overexpression, crude protein extract was blotted and probed with an anti-HosA antibody. A 55- and 77-kDa marker protein is shown (lane M). A wildtype strain was used to confirm the specificity of the antibody. *xylP*p repressing conditions (r, no xylose added) were used as negative control **(A)**. Phenotype of HosA overexpressing strains were subsequently assessed under *xylP*p inductive and repressive conditions on agar plates. hosAoe1 and 2, a *hosA*-complemented (*hosA*c), and a *hosA* deletion (Δ*hosA*) strain were grown for 48 h at 37°C on GMM with (i) or without (r) 1% xylose, before the colonies were compared to a wildtype strain **(B)**.

FIGURE S3 Heatmap of the expression levels of 70 confirmed or predicted key enzymes (polyketide synthases or non-ribosomal peptide synthetases) of secondary metabolite gene clusters in *hosA* mutants and wildtype grown at 37°C. The FPKM + 1 values were log10-transformed and genes were ranked based on their aberration in transcription in mutants versus wildtype of short-term (24 h) or long-term (60 h) cultures, respectively. The blue box indicates enzymes for which differential expression has been confirmed by Northern blot analysis. Blue arrowheads indicate 25 selected genes that were subsequently analyzed together with their adjacent coding sequences in **Supplementary Table S7**.

FIGURE S4 Subcellular localization of HosA and RpdA in HosA-GFP and RpdA-GFP expression strains. Strains were grown under 0.1% xylose to achieve moderate expression of the recombinant HDACs RpdA and HosA, respectively. DNA was stained with DAPI. RpdA was predominantly localized in the nucleus whereas HosA could be detected in both, the nucleus and the cytoplasm.

TABLE S1 Genotypes of fungal and bacterial strains used in this study. The lab name and the pseudonym of fungal strains used in this manuscript are specified. Genetic characteristic is indicated as follows: *hosA::argB* indicates the replacement of *hosA* by the auxotrophic marker gene encoding ornithine transcarbamylase (*argB*) used for selection of transformants, *xylpP:hosA:His:argB; argB2* refers to a copy of His-tagged *hosA* under control of the heterologous xylanase promoter (*xylP*p) randomly integrated into the genome in addition to endogenous *hosA.* For the generation of some mutants, a phleomycin resistance gene (*bleR*) was used instead of *argB* as selection marker. Strains H1 and TBF53.1 or TBF53.2 were used for sexual crosses to gain *hdaA/hosA* double knock out mutants, χ is indicating sexual crosses of the corresponding strains.

TABLE S2 Oligonucleotides used for qPCR and generation of probes. Primers for qPCR of the ChIP analysis are listed; three different regions (T1, T2, T3) in each of the corresponding SM cluster genes were used for quantification of precipitated DNA (left column). Primer pairs used to generate Dig-dUTP-labeled DNA probes for transcriptional analysis in Northern blots are shown (right column). PN, penicillin; OA, orsellinic acid; CC, cichorine; AC, aspercryptin.

TABLE S3 TopHat summary of mapped reads of each RNA-Seq sample. Table shows the amount of input reads (reached from Illumina HiSeq Single Read sequencing), successfully mapped reads and number of reads aligned multiple times to the reference genome.

TABLE S4 Genes represented in Venn-diagrams and used for FunCat classification ontology analysis. Listed genes are the result of filtering Cuffdiff results of 10.820 genes for Cuffdiff-significance, FDR (<0.05) and log2FC (≥2). Genes that are regarded as being differentially expressed between wildtype and *hosA* deletion mutant grown for 24 and 60 h are shown. Genes are divided into up- and down-regulated transcripts including those that are overlapping in both conditions.

TABLE S5 Collection of 70 confirmed or predicted genes encoding polyketide synthases or non-ribosomal peptide synthetases responsible for the biosynthesis of secondary metabolites. Detailed information about the type of enzyme, gene description, locus of the coding sequence in the genome and the compound, which is biosynthesized by the gene cluster is given (data sheet 1). Expression levels in wildtype and *hosA* mutants after 24 and 60 h of growth are compared (data sheet 2). Expression confirmed by Northern blot experiments are highlighted in gray.

TABLE S6 Selected Cuffdiff results of the orsellinic acid, aspercryptin, cichorine and penicillin gene clusters of *hosA* mutants grown for 24 and 60 h compared to wildtype. Secondary metabolite gene clusters (gray) flanked by five up- and five downstream genes are shown to confirm the specific regulation of the SM clusters. Sample 1 is compared to sample 2 including the corresponding FPKM value for both samples, the resulting log2fc, the fold change and the *p*-value.

TABLE S7 Confirmed or putative secondary metabolite gene clusters and adjacent genes. (Predicted) key enzymes such as polyketide synthases or non-ribosomal peptide synthetases of the respective SM clusters are highlighted in the data sheets in gray. Expression levels of the key synthases and at least 20 adjacent genes flanking both sides of each key enzymes were compared between wildtype and *hosA* mutants of short-term (24 h) and long-term (60 h) cultures. Relating to the log2FC, a cutoff of ±1.0 was applied considering the significance criteria (sig) of Cuffdiff.

TABLE S8 Results of FungiFun2 analysis performed with the FunCat classification ontology option. Genes listed in **Supplementary Table S4** were used as input IDs. For each comparison between wildtype and *hosA* mutant strains grown for 24 and 60 h, genes were divided into up- and downregulated categories. Category and gene-ID summary containing gene-ID, protein name, FunCat ID with description, and *p*-values are shown.
