# Erratum: A Class 1 Histone Deacetylase as Major Regulator of Secondary Metabolite Production in *Aspergillus nidulans*

**DOI:** 10.3389/fmicb.2018.02714

**Published:** 2018-11-09

**Authors:** Frontiers Production Office

**Affiliations:** Frontiers Production Office, Frontiers Media SA, Lausanne, Switzerland

**Keywords:** histone deacetylases, secondary metabolites, histone modifications and chromatin structure, transcription factors, filamentous fungi, Aspergillus, antifungals

Due to a production error in the Introduction, Paragraph 3, the class of enzymes referred to in the citation of Brosch et al. (2008), was incorrectly indicated as class 1 and should be class 3. In the same paragraph, the class of enzymes referred to in the citation of Graessle et al. (2000), was unintentionally removed and should be class 1. The publisher apologizes for this error. The original article has been updated.
