# Supplementary figures and images for: A Class 1 Histone Deacetylase as Major Regulator of Secondary Metabolite Production in Aspergillus nidulans
Source: Front Microbiol. 2018 Sep 19;9:2212. doi: 10.3389/fmicb.2018.02212 (PMC6156440; doi:10.3389/fmicb.2018.02212)

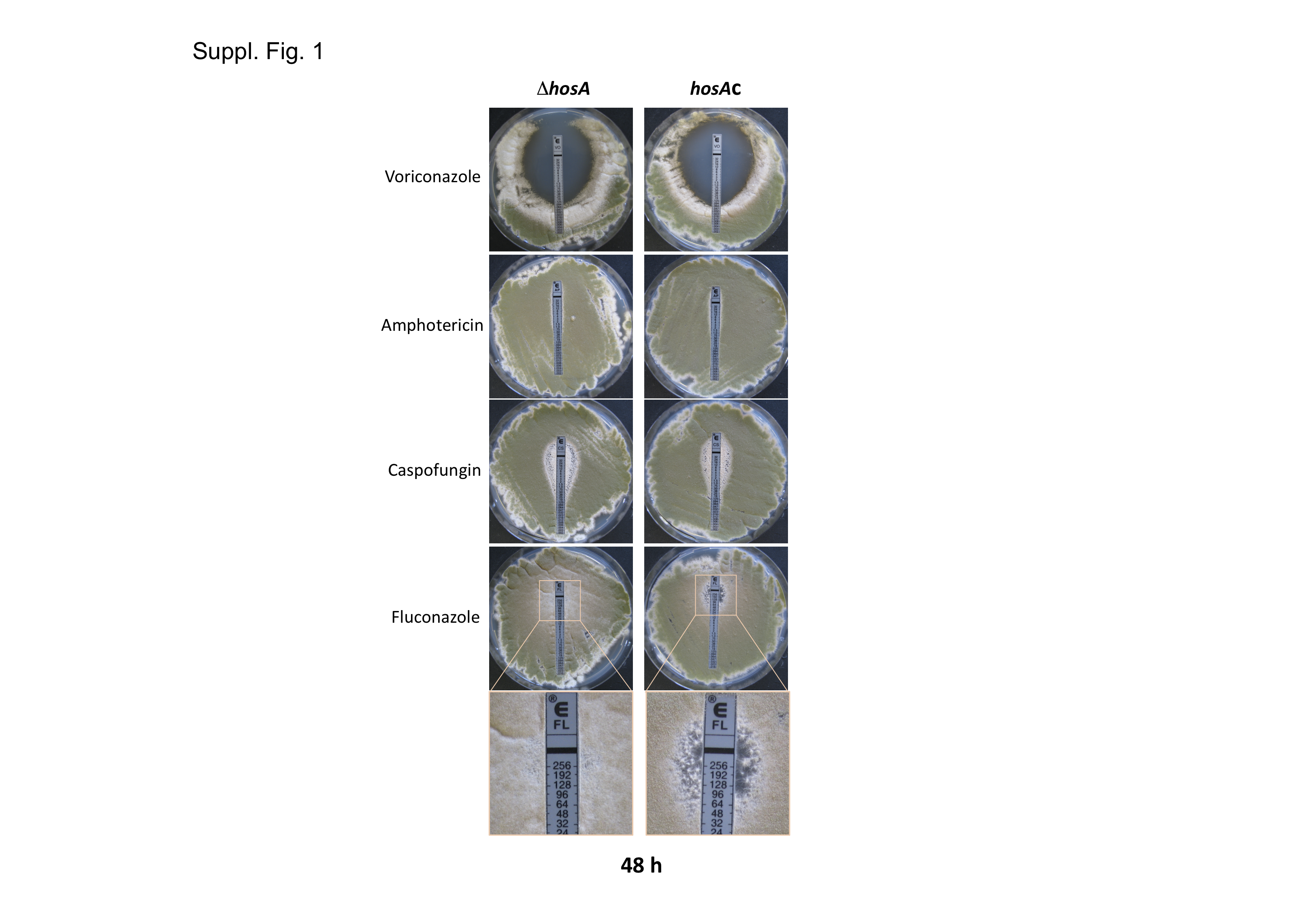

Supplement: FIGURE S1 — Sensibility of hosA mutants compared to complemented strains as tested by antifungigrams (ETEST). For sensibility testing against antifungal substances, ETEST strips loaded with an increasing concentration of the antifungals voriconazole, amphotericin, caspofungin, and fluconazole were applied to plates with spores of hosA deletion strains and wt according to the manufacturer’s instructions of the test-strips. After incubation for 24 (not shown) and 48 h at 37°C, the sensibility was evaluated (see magnification of the fluconazole strip below). [file Image_1.TIFF]

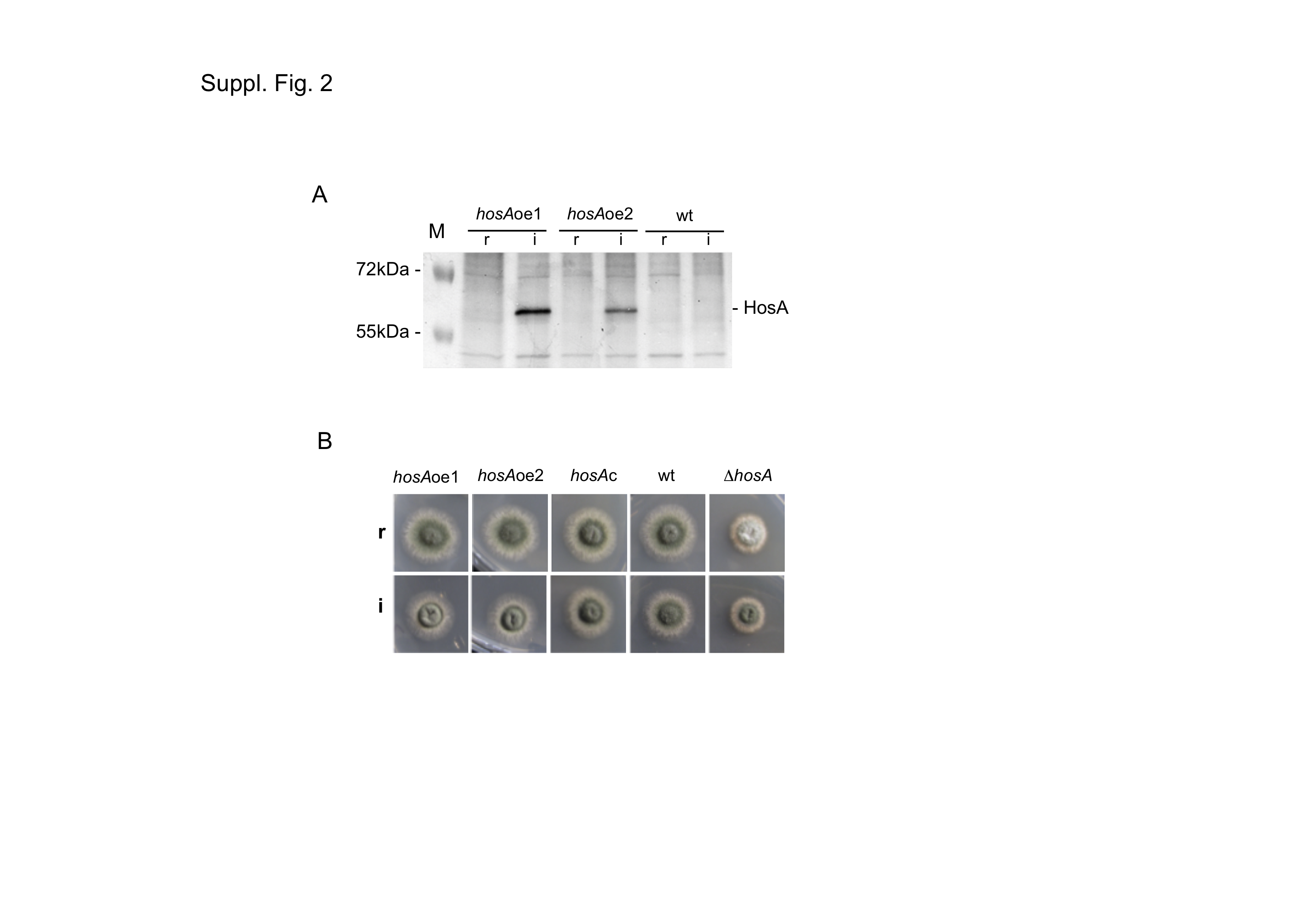

Supplement: FIGURE S2 — Phenotypical analysis of HosA overexpression strains. His tagged HosA was overexpressed in Aspergillus nidulans strains TBF117 (hosAoe1) and TBF122 (hosAoe2) under control of the heterologous xylanase promoter (xylPp) in submerged culture (GMM) supplemented with 1% xylose (i) for 48 h at 37°C. In order to verify HosA overexpression, crude protein extract was blotted and probed with an anti-HosA antibody. A 55- and 77-kDa marker protein is shown (lane M). A wildtype strain was used to confirm the specificity of the antibody. xylPp repressing conditions (r, no xylose added) were used as negative control (A). Phenotype of HosA overexpressing strains were subsequently assessed under xylPp inductive and repressive conditions on agar plates. hosAoe1 and 2, a hosA-complemented (hosAc), and a hosA deletion (ΔhosA) strain were grown for 48 h at 37°C on GMM with (i) or without (r) 1% xylose, before the colonies were compared to a wildtype strain (B). [file Image_2.TIFF]

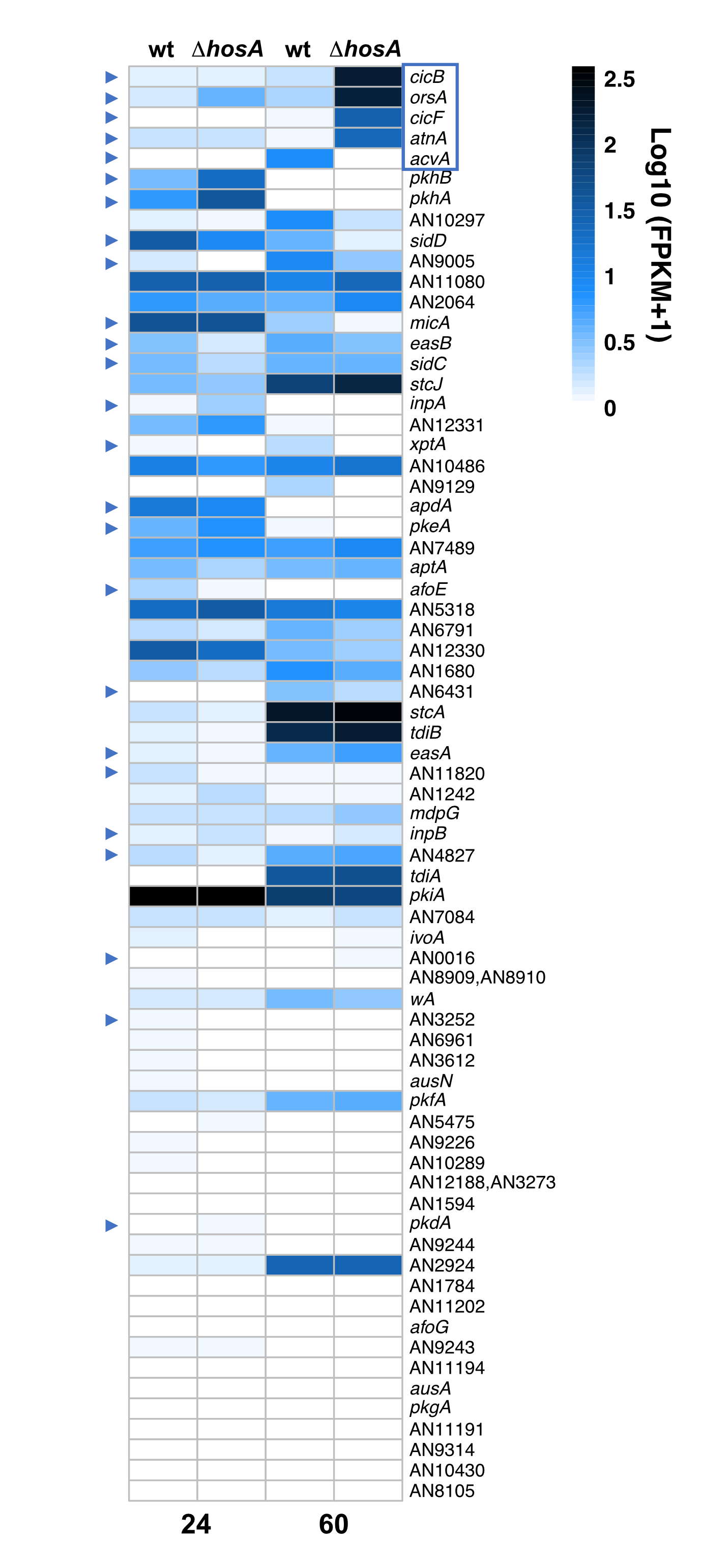

Supplement: FIGURE S3 — Heatmap of the expression levels of 70 confirmed or predicted key enzymes (polyketide synthases or non-ribosomal peptide synthetases) of secondary metabolite gene clusters in hosA mutants and wildtype grown at 37°C. The FPKM + 1 values were log10-transformed and genes were ranked based on their aberration in transcription in mutants versus wildtype of short-term (24 h) or long-term (60 h) cultures, respectively. The blue box indicates enzymes for which differential expression has been confirmed by Northern blot analysis. Blue arrowheads indicate 25 selected genes that were subsequently analyzed together with their adjacent coding sequences in Supplementary Table S7. [file Image_3.TIFF]

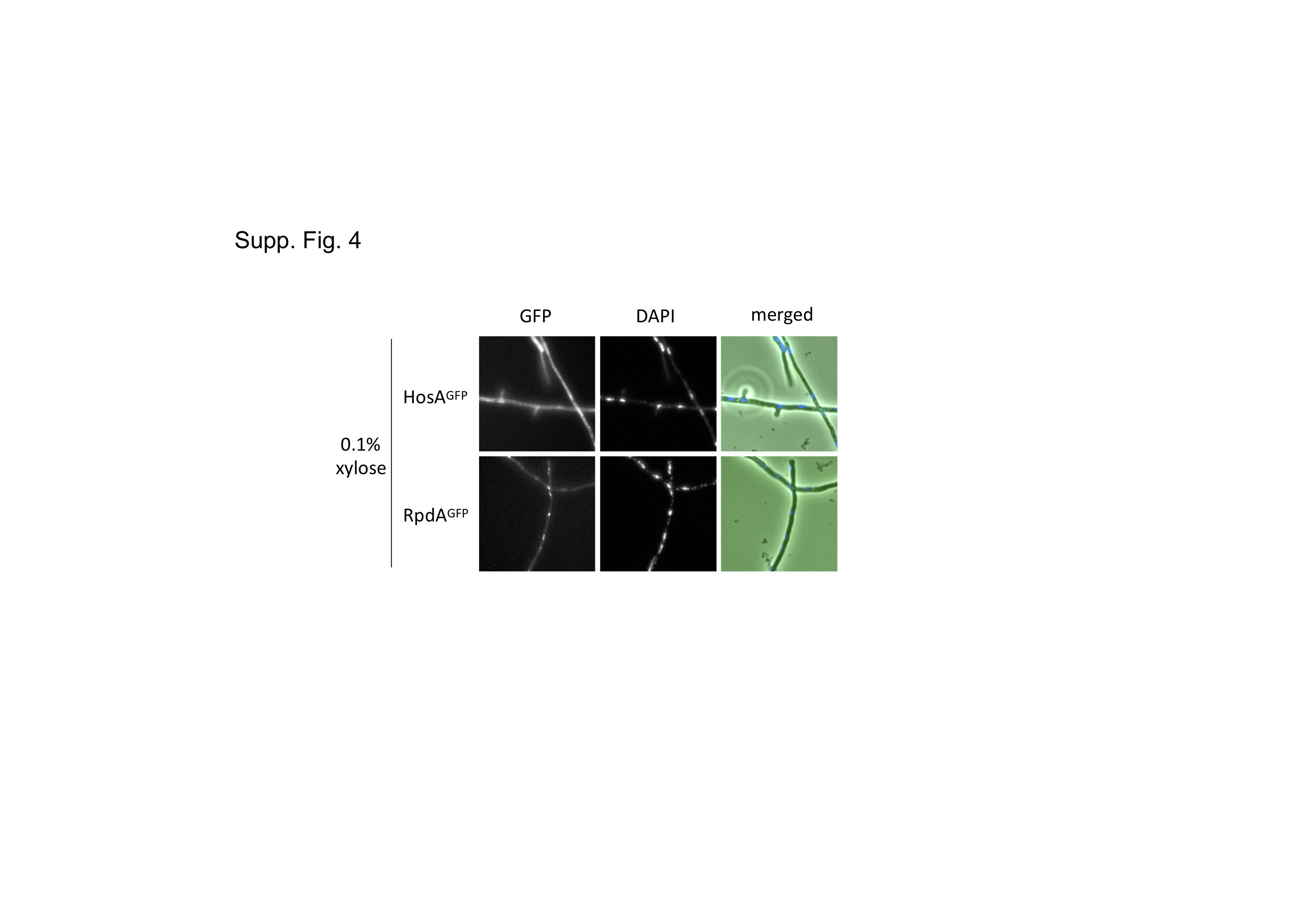

Supplement: FIGURE S4 — Subcellular localization of HosA and RpdA in HosA-GFP and RpdA-GFP expression strains. Strains were grown under 0.1% xylose to achieve moderate expression of the recombinant HDACs RpdA and HosA, respectively. DNA was stained with DAPI. RpdA was predominantly localized in the nucleus whereas HosA could be detected in both, the nucleus and the cytoplasm. [file Image_4.TIFF]
