# Supplementary material for: A Class 1 Histone Deacetylase as Major Regulator of Secondary Metabolite Production in Aspergillus nidulans
Source: Front Microbiol. 2018 Sep 19;9:2212. doi: 10.3389/fmicb.2018.02212 (PMC6156440; doi:10.3389/fmicb.2018.02212)
Supplement: TABLE S1 — Genotypes of fungal and bacterial strains used in this study. The lab name and the pseudonym of fungal strains used in this manuscript are specified. Genetic characteristic is indicated as follows: hosA::argB indicates the replacement of hosA by the auxotrophic marker gene encoding ornithine transcarbamylase (argB) used for selection of transformants, xylpP:hosA:His:argB; argB2 refers to a copy of His-tagged hosA under control of the heterologous xylanase promoter (xylPp) randomly integrated into the genome in addition to endogenous hosA. For the generation of some mutants, a phleomycin resistance gene (bleR) was used instead of argB as selection marker. Strains H1 and TBF53.1 or TBF53.2 were used for sexual crosses to gain hdaA/hosA double knock out mutants, χ is indicating sexual crosses of the corresponding strains. [file Table_1.pdf]

Supplementary Table 1: Genotypes of strains used in this study

Aspergillus nidulans

Strain	Ref. name study	Origin	Variation with regard to wildtype	Genotype	Reference
A89	wt		–	*biA1; argB2; veA1*	FGSC
TBF50.3	Δ*hosA*, *Δ*	A89	*hosA* deletion strain	*ΔhosA::argB; biA1; argB2; veA1; ArgB+*	this study
TBF53.1	ΔhosA, *Δ*	A89	*hosA* deletion strain	*ΔhosA::argB; biA1; argB2; veA1; ArgB+*	this study
TBFl2.3	*hosAc, c*	TBF53.1	*hosA* complemented strain	*ΔhosA::argB; biA1; argB2; veA1; hosA; bleR*	this study
TBFz6.1	*hosAc, c*	TBF53.1	*hosA* complemented strain	*ΔhosA::argB; biA1; argB2; veA1; hosA; bleR*	this study
TBF117	*hosAoe1*	A89	*hosA* overexpression strain	*xylPp:hosA:His:argB; argB2; biA1; veA1; ArgB+*	this study
TBF122	*hosAoe2*	A89	*hosA* overexpression strain	*xylPp:hosA:His:argB; argB2; biA1; veA1; ArgB+*	this study
TBFXT1.1	–	TBF53.1	*hosA*- TAP strain	*xylPp:hosA:TAP; ΔhosA::argB; biA1; argB2; veA1; bleR*	this study
TBFXT1.3	–	TBF53.1	*hosA*- TAP strain	*xylPp:hosA:TAP; ΔhosA::argB; biA1; argB2; veA1; bleR*	this study
A768	–	–	–	*riboB2; pyrG89; veA1; yA2; chaA1*	FGSC
H4	*Δ hdaA*	A768	*hdaA* deletion strain	*ΔhdaA::pyrG; riboB2; pyrG89; veA1; veA1; yA2; chaA1; PyrG+*	Tribus *et al.*, 2005
RBF115	*Δ hosA/hdaA*	TBF53.1 *X* H4	*hosA/hdaA deletion strain*	*ΔhosA::argB; ΔhdaA::pyrG; biA1; veA1; pyrG89?; argB2?; PyrG+; ArgB+*	this study
RBF117	*Δ hosA/hdaA*	TBF53.2 *X* H4	*hosA/hdaA deletion strain*	*ΔhosA::argB; ΔhdaA::pyrG; biA1; veA1; pyrG89?; argB2?; PyrG+; ArgB+*	this study
TIB54.1	RpdA-GFP	A89	*rpdA* -GFP strain	*xylPp:rpdA:GFP::argB; biA1; argB2; veA1; ArgB+*	this study
TBFGFP1.1	HosA-GFP	TBF53.1	*hosA*- GFP strain	*xylPp:hosA:GFP; ΔhosA::argB; biA1; arbB2; veA1; bleR*	this study
TBFGFP4.1	HosA-GFP	TBF53.1	*hosA*- GFP strain	*xylPp:hosA:GFP; ΔhosA::argB; biA1; arbB2; veA1; bleR*	this study

B) *Escherichia coli*

Strain	Reference
DH5α	Hanahan, 1983
GM99	Bale et al., 1979

B) *Kocuria rhizophila*

ATCC 9341	Tang and Gillevet, 2003
