# Supplementary material for: A Class 1 Histone Deacetylase as Major Regulator of Secondary Metabolite Production in Aspergillus nidulans
Source: Front Microbiol. 2018 Sep 19;9:2212. doi: 10.3389/fmicb.2018.02212 (PMC6156440; doi:10.3389/fmicb.2018.02212)
Supplement: TABLE S2 — Oligonucleotides used for qPCR and generation of probes. Primers for qPCR of the ChIP analysis are listed; three different regions (T1, T2, T3) in each of the corresponding SM cluster genes were used for quantification of precipitated DNA (left column). Primer pairs used to generate Dig-dUTP-labeled DNA probes for transcriptional analysis in Northern blots are shown (right column). PN, penicillin; OA, orsellinic acid; CC, cichorine; AC, aspercryptin. [file Table_2.PDF]

Supplementary Table 2: Primers used in this study

primers used for qPCR

PN-cluster *(ipnA)*

primer-name	sequence
ipnA_T1_for	TCCAGTCGAAGCACTGCCAA
ipnA_T1_rev	GGCACGGCTTGATGGTACG
ipnA_T2_for	GGCTCGATGTCCAAGCCAGT
ipnA_T2_rev	TTGCCGGCGAAGAACAGACG
ipnA_T3_for	GGAGACGACCAAGCAGCCAA
ipnA_T3_rev	TGCGAGAGTCGCTGCACATT

OA-cluster *(orsA)*

primer-name	sequence
orsA_T1_for	CTCTTGCCCGGCTTGCCTAA
orsA_T1_rev	TGGCTCACTTAATCATGGCTGGA
orsA_T2_for	GCAGGAGCGGGTCACCTTT
orsA_T2_rev	GGCACGCTCGAGTCCATCAA
orsA_T3_for	CGCCATCCGAAGCAGCAATG
orsA_T3_rev	ATGGGCCGTTGACACAGCAA

CC-cluster *(cicB)*

primer-name	sequence
cicB_T1_for	CACAAATGTCCGCCGCAACT
cicB_T1_rev	TTCGCTCGCGCTCTTACTCT
cicB_T2_for	CGAGGTCCGTACTCGGGTCT
cicB_T2 rev	GATTGCCGGACGCTTGAACG
cicB_T3_for	ACCTCTGCAGCAGCCATCAT
cicB_T3_rev	GGCAGACCCTCATTGGTGGA

AC-cluster *(atnA)*

primer-name	sequence
atnA_T1_for	GTCGCCTCTCCCATCTCACC
atnA_T1_rev	CAGGAGCGATGTCCGGTGT
atnA_T2_for	GCAGGCTCTGGAGGCGATATG
atnA_T2_rev	CATGGCCACAATCGGCAACG
atnA_T3_for	ACGAGCTGCCAGCGGATATT
atnA_T3_rev	CGGTGGTGTTGACGACAGACT

primers used for generation of probes

PN-cluster *(ipnA)*

primer-name	sequence
ipnAfor	AAAGATCGATGTTTCTCCCC
ipnArev	AGCACAACCGAGGCAAG

OA-cluster *(orsA)*

primer-name	sequence
orsAprobef	CTTTGACCACCGACTCTTC
orsAprobr	GCTTGAGAATTACTACACCC

CC-cluster *(cicB)*

primer-name	sequence
cicBFor	ATGGAGACCCTGGAAGAAG
cicBRev	CGTTTGGTAACTGGAGTCC

AC-cluster *(atnG)*

primer-name	sequence
atnG_N_for	ACGTACCTGTACTACCTTACCACAG
atnG_N_rev	GATACAGTAGACCAAGACGCAGA
