# Supplementary material for: A Class 1 Histone Deacetylase as Major Regulator of Secondary Metabolite Production in Aspergillus nidulans
Source: Front Microbiol. 2018 Sep 19;9:2212. doi: 10.3389/fmicb.2018.02212 (PMC6156440; doi:10.3389/fmicb.2018.02212)
Supplement: TABLE S3 — TopHat summary of mapped reads of each RNA-Seq sample. Table shows the amount of input reads (reached from Illumina HiSeq Single Read sequencing), successfully mapped reads and number of reads aligned multiple times to the reference genome. [file Table_3.PDF]

RNA-Seq – mapping statistics

sample	Input	Mapped	multiple alignments	mapped [%]
ΔhosA_24h_R1	62871246	59198665	134273 (0.2%), (279 have >20)	94.2
ΔhosA_24h_R2	59742591	56024133	128332 (0.2%), (324 have >20)	93.8
ΔhosA_24h_R3	54343447	51513596	119678 (0.2%), (192 have >20)	94.8
ΔhosA_60h_R1	64047299	60211555	123895 (0.2%), (38 have >20)	94.0
ΔhosA_60h_R2	40809785	38858680	75931 (0.2%), (19 have >20)	95.2
ΔhosA_60h_R3	74495986	65756678	157825 (0.2%), (58 have >20)	88.3
wt_24h_R1	59015992	55823995	136646 (0.2%), (275 have >20)	94.6
wt_24h_R2	56984174	53027141	116206 (0.2%), (173 have >20)	93.1
wt_24h_R3	52350566	49792199	104156 (0.2%), (294 have >20)	95.1
wt_60h_R1	59336341	46923897	104121 (0.2%), (53 have >20)	79.1
wt_60h_R2	65874110	56209451	136662 (0.2%), (46 have >20)	85.3
wt_60h_R3	64658325	56199117	149585 (0.3%), (60 have >20)	86.9
**total**	**714529862**	**649539107**		**90,90**
